# Repeated Administrations of Polyphenolic Extracts Prevent Chronic Reflexive and Non-Reflexive Neuropathic Pain Responses by Modulating Gliosis and CCL2-CCR2/CX3CL1-CX3CR1 Signaling in Spinal Cord-Injured Female Mice

**DOI:** 10.3390/ijms26073325

**Published:** 2025-04-02

**Authors:** Anna Bagó-Mas, Andrea Korimová, Karolína Bretová, Meritxell Deulofeu, Enrique Verdú, Núria Fiol, Petr Dubový, Pere Boadas-Vaello

**Affiliations:** 1 Research Group of Clinical Anatomy, Embryology and Neuroscience (NEOMA), Department of Medical Sciences, University of Girona, 17071 Girona, Catalonia, Spain; anna.bago@uvic.cat (A.B.-M.); meritxell.deulofeu@hipra.com (M.D.); enric.verdu@udg.edu (E.V.); 2 Division of Neuroanatomy, Department of Anatomy, Faculty of Medicine, Masaryk University, 62500 Brno, Czech Republic; andrea.korimova@med.muni.cz (A.K.); karolina.bretova@med.muni.cz (K.B.); 3 Department of Chemical Engineering, Agriculture and Food Technology, Polytechnic School, University of Girona, 17003 Girona, Catalonia, Spain; nuria.fiol@udg.edu

**Keywords:** spinal cord injury, chronic neuropathic pain, neuroinflammation, polyphenols, glia, chemokines

## Abstract

Neuropathic pain after spinal cord injury lacks any effective treatments, often leading to chronic pain. This study tested whether the daily administration of fully characterized polyphenolic extracts from grape stalks and coffee could prevent both reflexive and non-reflexive chronic neuropathic pain in spinal cord-injured mice by modulating the neuroimmune axis. Female CD1 mice underwent mild spinal cord contusion and received intraperitoneal extracts in weeks one, three, and six post-surgery. Reflexive pain responses were assessed weekly for up to 10 weeks, and non-reflexive pain was evaluated at the study’s end. Neuroimmune crosstalk was investigated, focusing on glial activation and the expression of CCL2/CCR2 and CX3CL1/CX3CR1 in supraspinal pain-related areas, including the periaqueductal gray, rostral ventromedial medulla, anterior cingulate cortex, and amygdala. Repeated treatments prevented mechanical allodynia and thermal hyperalgesia, and also modulated non-reflexive pain. Moreover, they reduced supraspinal gliosis and regulated CCL2/CCR2 and CX3CL1/CX3CR1 signaling. Overall, the combination of polyphenols in these extracts may offer a promising pharmacological strategy to prevent chronic reflexive and non-reflexive pain responses by modifying central sensitization markers, not only at the contusion site but also in key supraspinal regions implicated in neuropathic pain. Overall, these data highlight the potential of polyphenolic extracts for spinal cord injury-induced chronic neuropathic pain.

## 1. Introduction

Traumatic spinal cord injury (SCI) represents a profound neurological condition that alters lives and carries significant socioeconomic consequences for patients and their caregivers. While recent advancements in medical care have notably enhanced the stabilization and survival rates, progress in treatment options aimed at improving neurological outcomes remains limited [1]. One prominent consequence of spinal cord injury is the development of neuropathic pain [2,3], which ranges from 34% to 83% in SCI-injured patients [4,5] and dramatically affects their quality of life [6,7]. Current treatments for neuropathic pain offer only moderate efficacy and are associated with side effects that limit their utility [8]. The lack of suitable treatments for neuropathic pain can lead to the development of chronic SCI-induced pathological pain, which underscores the urgent need for alternative therapeutic strategies to prevent central neuropathic pain (CNP) development and its progression to chronic pain.

Emerging evidence suggests that the activation of glial cells leads to increased production of proinflammatory mediators, creating neuroimmune crosstalk in the central nervous system, and such a neuroinflammatory state has been proven to be a fundamental mechanism in pathological pain development and its transition to chronic pain [9,10]. That is, the role of cytokines and chemokines in relation to the communication of the neuroinflammatory state between glia and neurons would have a pivotal role in SCI-induced neuropathic pain [11,12]. Thus, new pharmacological strategies targeting the modulation of neuroinflammatory states would potentially be suitable treatments to prevent the development of chronic neuropathic pain.

In this line, it has been shown over recent decades that natural polyphenolic compounds can exert anti-inflammatory effects in several health issues [13], including in pathological pain conditions [14,15]. When focusing on central neuropathic pain, studies demonstrated that polyphenols could prevent the development of acute SCI-induced neuropathic pain in preclinical models by modulating neuroinflammation [16,17,18]. Recent studies of our own specifically demonstrated that natural polyphenolic extracts exert preventive effects after SCI by downregulating the expression of neuron–glia crosstalk-related biomolecules such as CX3CL1/CX3CR1, CCL2/CCR2 [17]. However, it is important to note that these promising results were primarily observed during the acute phase of SCI. It is also noteworthy that chronic neuropathic pain, in addition to reflexive pain responses like thermal hyperalgesia and mechanical allodynia, encompasses emotional or affective disturbances known as non-reflexive pain responses. These disturbances may be associated with dysfunction or neuroinflammatory processes in the supraspinal structures that play crucial roles in these behavioral components [19,20,21]. Hence, novel pharmacological strategies targeting neuroinflammation in both the spinal cord and supraspinal structures should likely be highly effective in preventing both reflexive and non-reflexive chronic pain responses.

Considering the demonstrated efficacy of polyphenolic natural extracts in modulating both reflexive and non-reflexive pain responses in acute phase pathological pain models [16,17,22], further investigation into their potential for preventing chronic pain is essential. This is especially crucial in understanding their impact on supraspinal alterations and non-reflexive pain responses, which have been highlighted as significant factors in the chronic phase of spinal cord injury (SCI) [21,23,24,25]. To address this, our study was designed to assess whether treatment with these fully characterized polyphenolic extracts (GSE–grape stalk extract and CE–coffee extract) [17] could effectively modulate both reflexive and non-reflexive chronic neuropathic pain responses over time, extending into the chronic phase of injury. Recognizing the temporal progression of SCI in mice, with immediate and acute phases typically spanning the initial 2–3 weeks post-injury [26], followed by an intermediate phase lasting from 3 to 6 weeks post-injury [27], and the onset of the chronic phase usually occurring around 6–8 weeks post-injury [28], our study implemented an innovative protocol. This protocol involved the repeated administration of polyphenolic extracts during the first week post-injury to prevent the development of CNP, and additional administrations during the third and sixth weeks post-injury to reinforce these preventive effects.

## 2. Results

### 2.1. Repeated Administration of GSE15 or CE10 During the First, Third, and Sixth Weeks Following Mild Spinal Cord Injury Prevents the Development of Both Mechanical Allodynia and Thermal Hyperalgesia

The weekly doses of GSE and CE administered during the first, third, and sixth weeks after SCI were 15 mg/kg (GSE15) and 10 mg/kg (CE10), respectively. These doses have been identified as the most effective in preventing the development of central neuropathic pain during the acute SCI phase [17].

**Figure 1:**
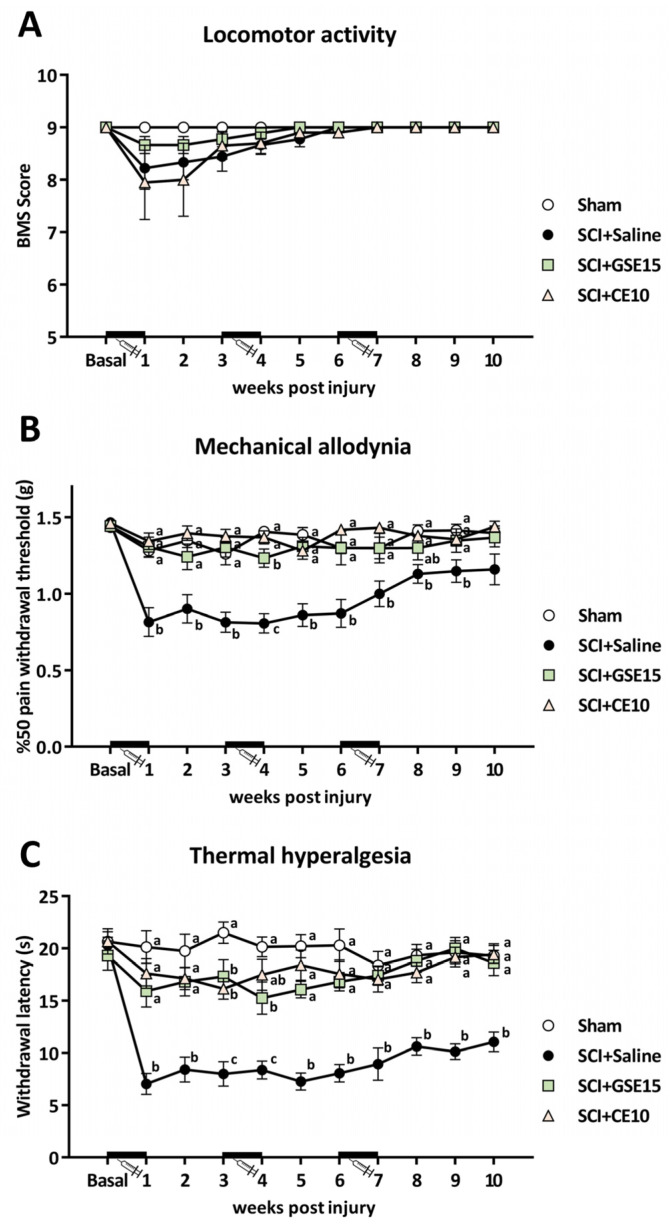
Time-course assessment of (**A**) locomotor activity, (**B**) mechanical allodynia and (**C**) thermal hyperalgesia during the acute, intermediate and chronic phase of SCI-induced neuropathic pain after GSE15 and CE10 treatments in the first, third and sixth week post-injury. Each point and vertical line represent the mean ± SEM. Treatment administration weeks (basal to 1 wpi, 3 to 4 wpi and 6 to 7 wpi) are highlighted with a thick black line. a–c: groups not sharing a letter are significantly different, *p* < 0.05, by post hoc test. Experimental groups: sham (*n* = 8), SCI + saline (*n* = 9), SCI + GSE15 (*n* = 9), SCI + CE10 (*n* = 10).

Locomotor activity was assessed to ensure it did not interfere with reflexive-pain response evaluation. Data did not follow a normal distribution (all *p* values < 0.001, Kolmogorov–Smirnov test). The Friedman test revealed significant differences over the experimental period (*p* < 0.001), but the Kruskal–Wallis test found no significant differences between groups post-injury (all *p* values > 0.268) (Figure 1A). Notably, alterations were limited to paw position, indicating mice maintained free movement without paralysis or major impairment in locomotor functions.

Referring to mechanical allodynia, the Kolmogorov–Smirnov normality test showed that the data did not follow a normal distribution (*p* < 0.05). Friedman’s test indicated significant differences in the distribution of the data (*p* < 0.001), and a subsequent Kruskal–Wallis test revealed significant differences between groups at all weeks post-injury (all *p* values < 0.038), except at 10 wpi (*p* = 0.128). Specifically, post hoc Mann–Whitney U tests demonstrated a significant increase in mechanical sensitivity in the SCI + saline group compared with the sham group up to 9 wpi (all *p* values < 0.05). Although the SCI + saline group showed a significant decrease in paw withdrawal mechanical thresholds when compared with sham up to 9 wpi, an upward trend in these thresholds was observed from 7 wpi until the end of the experimental period. As for polyphenolic treatments, both GSE15 and CE10 significantly prevented mechanical allodynia development after SCI up to 9 wpi. While the SCI + CE10 group did not significantly differ from sham at any experimental time point, the SCI + GSE15 group showed significant differences compared with sham at 4 wpi (*p* = 0.031). Moreover, at 8 wpi, due to the loss of mechanical sensitivity in the SCI + saline group, SCI animals treated with GSE15 showed no significant differences from either SCI + saline or sham (Figure 1B).

In contrast to mechanical allodynia, the thermal hyperalgesia data followed a normal distribution (*p* > 0.05), as indicated by the Kolmogorov–Smirnov test. Therefore, a MANOVA was performed, revealing the significant main effects of the treatment (*p* < 0.001) and week (*p* = 0.022), as well as a significant week × treatment interaction (*p* = 0.001). Subsequent ANOVA indicated significant group differences at all post-injury weeks from 1 to 10 (all *p* values < 0.001). Specifically, the SCI + saline group showed a significant decrease in paw withdrawal latency to thermal stimulation compared to the sham group. Post hoc analyses also revealed that both GSE15 and CE10 treatments significantly prevented the development of thermal hyperalgesia in SCI animals from 1 to 10 wpi. Notably, the SCI + CE10 group did not differ significantly from sham at any post-injury week except 3 wpi, while the SCI + GSE15 group showed no significant difference from sham except at 3 and 4 wpi (Figure 1C).

Altogether, these findings indicate that both polyphenolic treatments effectively prevented the development of thermal hyperalgesia for up to 10 weeks post-SCI, extending into the chronic phase of the injury. Additionally, both polyphenolic extracts demonstrated a significant modulation of mechanical allodynia, persisting until week 9 when this response was still evident in the SCI animals. These results cannot be attributable to locomotor disturbances since no alterations were found.

### 2.2. Repeated GSE15 or CE10 Administration During the First, Third and Sixth Week Post-Injury Modulates Non-Reflexive Pain Behaviors Induced by Mild Spinal Cord Injury at 10 Weeks Post-Injury

**Figure 2:**
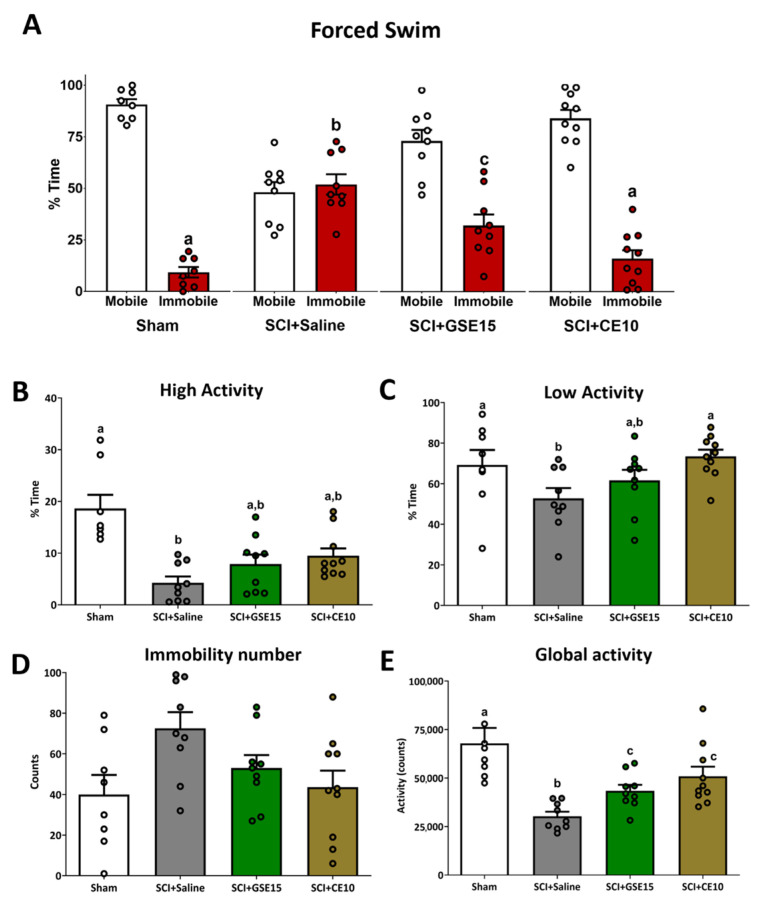
Forced swim test at the chronic phase of SCI-induced neuropathic pain after GSE15 and CE10 treatments in the first, third and sixth week post-injury. Histograms represent (**A**) the percentage of total time spent in mobility and immobility, (**B**) the percentage of high activity, (**C**) the percentage of low activity, as well as the total counts of (**D**) immobility and (**E**) global activity. Results are represented as mean ± SEM. a–c: groups not sharing a letter are significantly different, *p* < 0.05, by post hoc test. Experimental groups: sham (*n* = 8), SCI + saline (*n* = 9), SCI + GSE15 (*n* = 9), SCI + CE10 (*n* = 10).

Depressive-like behavior was evaluated by the forced swimming test [16,22]. The Kolmogorov–Smirnov normality test confirmed normal distribution for the percentage of mobility and immobility time, and number of immobility and low activity (all *p* values > 0.05). However, the high and global activity parameters did not follow a normal distribution (*p* < 0.05). ANOVA analysis revealed significant group differences for the percentage of immobility time (*p* < 0.001). SCI + saline animals spent more time immobile compared to sham and SCI animals treated with GSE15 or CE10 (*p* < 0.05). CE10 reduced immobility time to sham levels, while GSE15 significantly decreased it without reaching sham values (Figure 2A).

Significant group differences were also found in global activity (Kruskal–Wallis, *p* < 0.05). Sham animals exhibited significantly higher overall activity than SCI + saline animals (*p* < 0.05). Importantly, this immobility in the SCI + saline group cannot be attributed to locomotor deficits, as the BMS test did not reveal any significant alterations in locomotor function. Although GSE15 and CE10 increased global activity in SCI animals, they did not reach sham levels (Figure 2E). In terms of high and low activity time, significant group differences were detected (Kruskal–Wallis, *p* = 0.016 and *p* = 0.04). Sham animals spent more time in both high and low activity compared to SCI + saline (*p* < 0.05). SCI animals treated with GSE15 or CE10 spent more time in high activity but did not significantly differ from SCI + saline or sham (*p* > 0.05). However, SCI animals treated with GSE15 or CE10 spent more time in low activity compared to SCI + saline, with differences observed between SCI + CE10 and sham (Figure 2B,C). Immobility number did not differ significantly between groups (ANOVA, *p* = 0.113) (Figure 2D).

**Figure 3:**
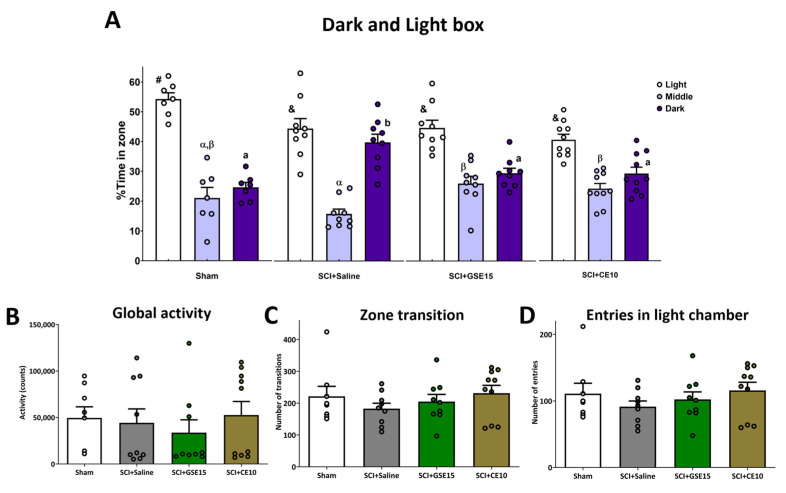
Light and dark box test at the chronic phase of SCI-induced neuropathic pain after GSE15 and CE10 treatments in the first, third and sixth week post-injury. Histograms represent (**A**) the percentage of total time spent in dark and light zones, (**B**) the counts of global activity, (**C**) the number of transition between dark and light zones, and (**D**) the number of entries in the light zone. Results are represented as mean ± SEM. #-&/α-β/a-b: groups not sharing a symbol/letter are significantly different, *p* < 0.05, by post hoc test. Experimental groups: sham (*n* = 8), SCI + saline (*n* = 9), SCI + GSE15 (*n* = 9), SCI + CE10 (*n* = 10).

Anxiety was evaluated by dark and light (DL) [21] and open field (OF) tests [29]. In the dark and light (DL) test, parameters mostly followed a normal distribution (*p* > 0.05), except for global activity (*p* < 0.001). Significant group differences were found in the time spent in the light (*p* = 0.017), dark (*p* < 0.001), and intermediate zones (*p* = 0.008). Sham animals spent more time in the light and less in the dark compared to SCI + saline. SCI animals treated with GSE15 or CE10 spent more time in the intermediate zone, approaching sham levels (Figure 3A). Although a trend was observed, no significant group differences were found in zone transition (*p* = 0.504) or entries in the light chamber (*p* = 0.501). However, GSE15 and CE10-treated SCI animals exhibited more transitions and entries than non-treated SCI animals (Figure 3C,D). Global activity did not differ significantly between the groups (*p* > 0.578), suggesting that the transition parameters were not influenced by drug-induced motor effects (Figure 3B). In other words, the observed behavioral results are unlikely to stem from locomotor disturbances, given that no differences in transitions or global activity were detected in the dark/light box test. In the open field test, most parameters followed a normal distribution (*p* > 0.05), except for latency to entry in the middle and center zones (*p* < 0.01).

**Figure 4:**
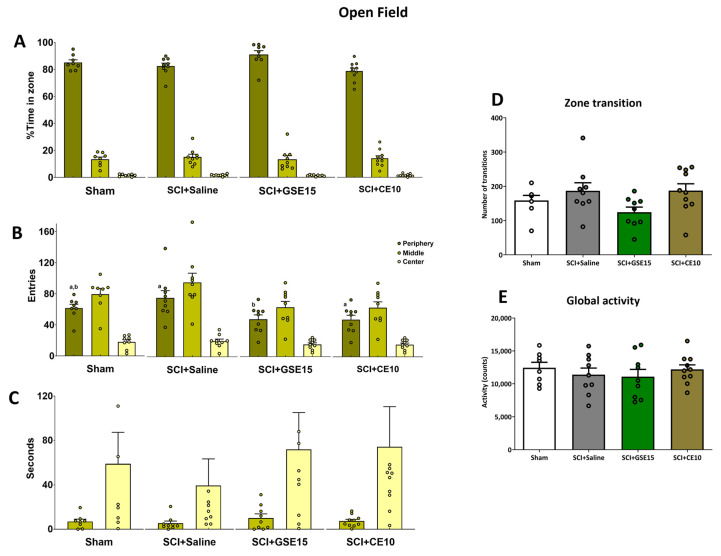
Open field test at the chronic phase of SCI-induced neuropathic pain after GSE15 and CE10 treatments in the first, third and sixth week post-injury. Histograms represent (**A**) the percentage of total time spent in the periphery, middle, and center zones, (**B**) the number of entries into these zones, (**C**) the time spent in these zones, (**D**) the number of transitions between zones, and (**E**) the global counts during activity. Results are represented as mean ± SEM, a–b: groups not sharing a letter are significantly different, *p* < 0.05, by post hoc test. Experimental groups: sham (*n* = 8), SCI + saline (*n* = 9), SCI + GSE15 (*n* = 9), SCI + CE10 (*n* = 10).

Subsequent analyses showed no significant group differences in most parameters, including percentage of time spent in zones, latency to entry, entries to zones, zone transition, and global activity (*p* > 0.05), except for entries to the peripheral zone (*p* = 0.033) (Figure 4A–E). Specifically, SCI + GSE15 had fewer entries in the periphery compared to SCI + saline and SCI + CE10 but did not significantly differ from sham (Figure 4C). Overall, while some anxiety-related parameters showed trends or significance, collectively, the dark and light and open field tests might suggest no significant development of general anxiety in the chronic phase of SCI.

**Figure 5:**
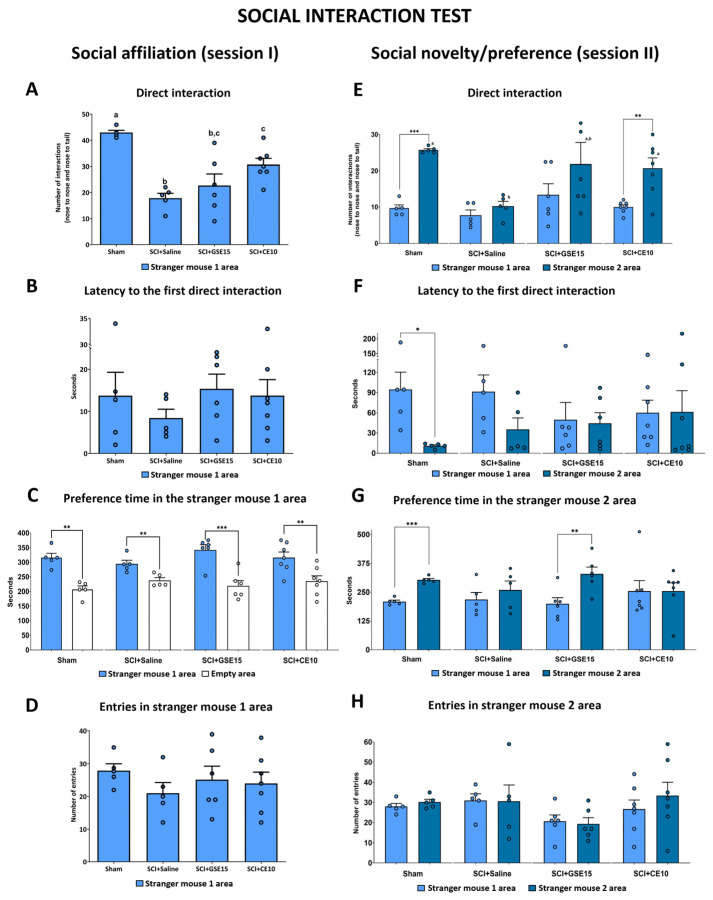
Social interaction test at the chronic phase of SCI-induced neuropathic pain after GSE15 and CE10 treatments in the first, third and sixth week post-injury. Histograms represent the number of direct interactions, the latency to the first interaction, the total time in the stranger mouse area, and the number of entries into the stranger mouse area during (**A**–**D**) session 1 and (**E**–**H**) session two. Results are represented as mean ± SEM, a–c: groups not sharing a letter are significantly different, *p* < 0.05, by post hoc test. Intra-groups significant differences: *** *p* < 0.001, ** *p* < 0.01, * *p* < 0.05. Experimental groups: sham (*n* = 5), SCI + saline (*n* = 5), SCI + GSE15 (*n* = 6), SCI + CE10 (*n* = 7).

Regarding the social interaction test, this involved two trials: social affiliation (session I) and social novelty/preference (session II). In session I, we found significant differences between the groups in direct interaction (*p* = 0.003). Compared to sham, untreated spinal cord-injured mice (SCI + saline) exhibited a notable decrease in direct interaction with stranger mouse 1. While the GSE15 treatment led to an increase in interactions between SCI mice and the stranger mouse, the SCI + GSE15 group did not show significant differences compared to the SCI + saline group. Conversely, CE10 treatment significantly boosted the number of interactions between SCI mice and the stranger mouse, though it did not quite reach the interaction levels of the sham group (Figure 5A). The other parameters we analyzed in session 1 did not show significant group differences (Figure 5B–D), and all groups spent more time in the stranger mouse 1 area than in the empty area (Figure 5C).

Moving to session II, we observed significant group differences in direct interaction with stranger mouse 2 (*p* = 0.043). Sham animals engaged in significantly more interactions with stranger mouse 2 compared to SCI + saline animals. While the GSE15 treatment led to increased interactions between SCI mice and stranger mouse 2, it did not show significant differences compared to either the SCI + saline or sham group. In contrast, the CE10 treatment significantly increased the number of interactions between SCI mice and stranger mouse 2 to levels comparable with the sham group. In addition, our analysis revealed that only the sham group and the SCI + CE10 group showed a significantly higher number of interactions with stranger mouse 2 compared to stranger mouse 1 (*p* < 0.001 and *p* = 0.008, respectively). However, we found no significant group differences in the number of direct interactions with stranger mouse 1 (*p* = 0.174) (Figure 5E). As with session I, the other parameters analyzed in session II did not show significant group differences (Figure 5F–H). Specifically, only the sham and SCI + GSE15 groups showed a preference for spending more time in the stranger mouse 2 area than in the stranger mouse 1 area (*p* < 0.001 and *p* = 0.009, respectively) (Figure 5G).

### 2.3. Repeated GSE15 or CE10 Administration During the First, Third and Sixth Week Post-Injury Reduces Astrogliosis and Modulates CCL2/CCR2 and CX3CL1/CX3CR1 Signaling in PAG and RVM at 10 Weeks Post-SCI

The periaqueductal grey (PAG) and rostral ventromedial medulla (RVM) play pivotal roles in modulating nociception [30]. While the PAG is known to induce robust analgesic effects through the activation of endogenous opioid systems [31], RVM has dual functions in either facilitating or inhibiting nociceptive signals, serving as a crucial relay in the descending regulation of pain [32]. PAG demonstrates a columnar functional organization, with ventrolateral (vlPAG) and dorsolateral (dlPAG) columns exhibiting varying levels of antinociceptive effects. Considering the distinct functions of PAG subregions in pain regulation [33,34], we investigated the levels of gliosis markers (GFAP and IBA1), chemokines CCL2 and CX3CL1, along with their respective receptors (CCR2 and CX3CR1), and CatS, which is responsible for the proteolytic release of soluble CX3CL1 [35], separately in the dlPAG and vlPAG columns and their potential association with changes in the RVM.

**Figure 6:**
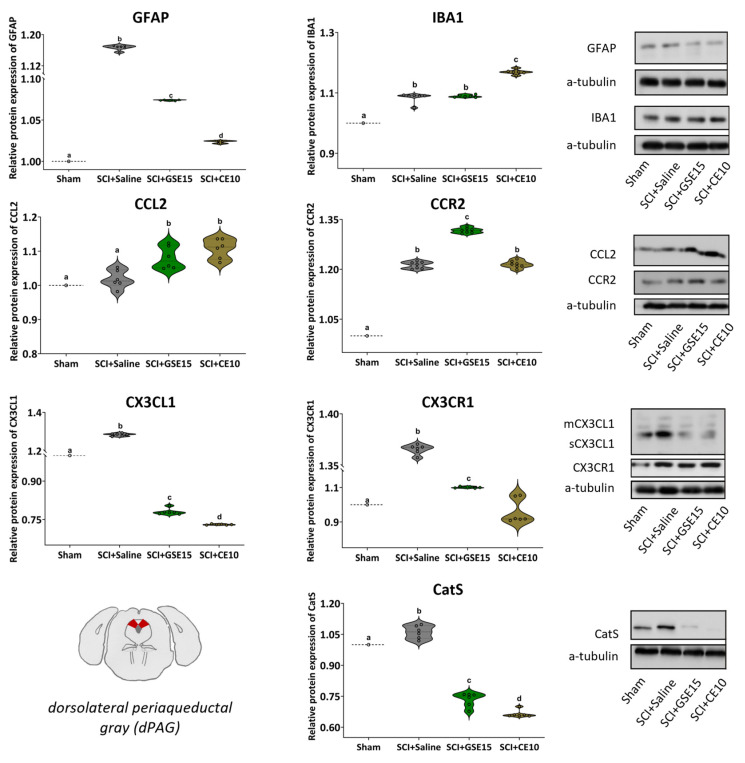
Expression of GFAP, IBA1, CCL2, CCR2, CX3CL1, CX3CR1 and CatS in dPAG at the chronic phase of SCI-induced neuropathic pain after GSE15 and CE10 treatments in the first, third and sixth week post-injury. Protein expression was normalized to a-tubulin. Data are expressed as a relative percentage with respect to sham group (mean ± SEM). a–d: Groups not sharing a letter showed significant differences, *p* < 0.05. Experimental groups: sham (GFAP *n* = 6; IBA1 *n* = 6; CCL2 *n* = 6; CCR2 *n* = 7; CX3CL1 *n* = 6; CX3CR1 *n* = 6; CatS *n* = 6), SCI + saline (GFAP *n* = 6; IBA1 *n* = 6; CCL2 *n* = 6; CCR2 *n* = 7; CX3CL1 *n* = 6; CX3CR1 *n* = 6; CatS *n* = 6), SCI + GSE15 (GFAP *n* = 6; IBA1 *n* = 6; CCL2 *n* = 6; CCR2 *n* = 7; CX3CL1 *n* = 6; CX3CR1 *n* = 6; CatS *n* = 6), SCI + CE10 (GFAP *n* = 6; IBA1 *n* = 6; CCL2 *n* = 6; CCR2 *n* = 7; CX3CL1 *n* = 6; CX3CR1 *n* = 6; CatS *n* = 6). Figure S1 of Supporting Information file shows full-length blot images corresponding to the cropped Western blot presented in the figure.

The Shapiro–Wilk normality test revealed that none of the markers analyzed in either dlPAG or vlPAG followed a normal distribution (all *p* values < 0.05). Further Kruskal–Wallis tests showed significant differences in protein levels between groups (all *p* values < 0.001). In dlPAG, the levels of all protein markers were increased in SCI saline-treated animals compared to sham animals (all *p* values < 0.01), except CCL2, which exhibited similar levels in both sham and SCI + saline groups (*p* = 0.065). Both GSE15 and CE10 treatments reduced GFAP, CX3CL1/CX3CR1, and CatS levels in dlPAG compared to saline-treated SCI animals (all *p* values < 0.01). In addition, GSE15 decreased CX3CL1 and CatS levels below those in the sham group (*p* values < 0.01). Although GSE15 decreased GFAP and CX3CR1 levels, they remained significantly higher than those in the sham group (*p* < 0.01). CE also reduced CX3CL1 and CatS levels below sham levels (*p* values < 0.01), but the reduced level of CX3CR1 was similar to that of the sham group (*p* = 0.394). Although CE reduced astrogliosis compared to the SCI-saline group, the level of GFAP expression remained significantly higher than that in the sham group (*p* < 0.01). Animals treated with either GSE15 or CE10 exhibited increased CCL2 expression in dlPAG compared to saline-treated animals (*p* < 0.01). Similarly, CE10-treated animals showed the increased level of IBA1 compared to SCI + saline animals (*p* < 0.01), whereas GSE15-treated animals showed IBA1 levels similar to SCI + saline animals (*p* = 0.699) (Figure 6).

**Figure 7:**
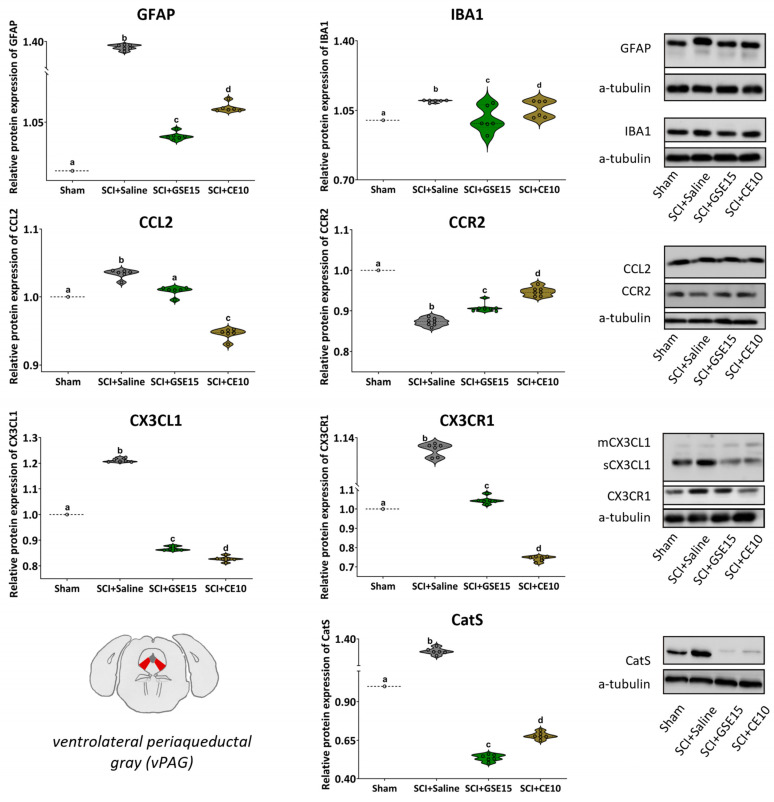
Expression of GFAP, IBA1, CCL2, CCR2, CX3CL1, CX3CR1 and CatS in vPAG at the chronic phase of SCI-induced neuropathic pain after GSE15 and CE10 treatments in the first, third and sixth week post-injury. Protein expression was normalized to a-tubulin. Data are expressed as a relative percentage with respect to sham group (mean ± SEM). a–d: Groups not sharing a letter showed significant differences, *p* < 0.05. Experimental groups: sham (GFAP *n* = 6; IBA1 *n* = 6; CCL2 *n* = 6; CCR2 *n* = 7; CX3CL1 *n* = 6; CX3CR1 *n* = 6; CatS *n* = 6), SCI + saline (GFAP *n* = 6; IBA1 *n* = 6; CCL2 *n* = 6; CCR2 *n* = 7; CX3CL1 *n* = 6; CX3CR1 *n* = 6; CatS *n* = 6), SCI + GSE15 (GFAP *n* = 6; IBA1 *n* = 6; CCL2 *n* = 6; CCR2 *n* = 7; CX3CL1 *n* = 6; CX3CR1 *n* = 6; CatS *n* = 6), SCI + CE10 (GFAP *n* = 6; IBA1 *n* = 6; CCL2 *n* = 6; CCR2 *n* = 7; CX3CL1 *n* = 6; CX3CR1 *n* = 6; CatS *n* = 6). Figure S1 of Supporting Information file shows full-length blot images corresponding to the cropped Western blot presented in the figure.

In vlPAG, levels of all molecular markers were increased in SCI saline-treated animals compared to sham animals (all *p* values < 0.01), except for CCR2 level, which was lower in saline-treated animals compared to sham animals (*p* < 0.01). Both GSE15 and CE10 treatments reduced GFAP, IBA1, CCL2, CX3CL1, CX3CR1, and CatS levels below those observed in the SCI + saline group (all *p* values < 0.01). GSE15 reduced CX3CL1 and CatS levels below sham levels (*p* values < 0.01), and IBA1 and CCL2 levels similar to those in the sham group (*p* values > 0.05), while reduced levels of GFAP and CX3CR1 did not reach sham levels (*p* values < 0.01). CE10 treatment reduced levels of CCL2, CX3CL1, CX3CR1, and CatS below sham levels (all *p* values < 0.01), and modulated both astrogliosis and microgliosis in vlPAG, but with GFAP and IBA1 levels remaining significantly higher than those in the sham group (*p* < 0.01) (Figure 7).

**Figure 8:**
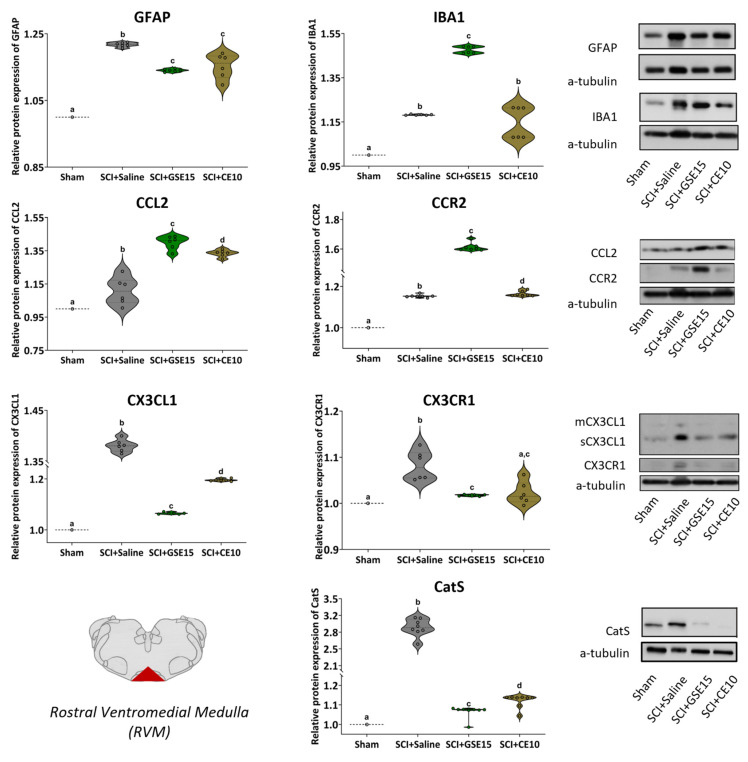
Expression of GFAP, IBA1, CCL2, CCR2, CX3CL1, CX3CR1 and CatS in RVM at the chronic phase of SCI-induced neuropathic pain after GSE15 and CE10 treatments in the first, third and sixth week post-injury. Protein expression was normalized to a-tubulin. Data are expressed as a relative percentage with respect to sham group (mean ± SEM). a–d: Groups not sharing a letter showed significant differences, *p* < 0.05. Experimental groups: sham (GFAP *n* = 6; IBA1 *n* = 6; CCL2 *n* = 6; CCR2 *n* = 7; CX3CL1 *n* = 6; CX3CR1 *n* = 6; CatS *n* = 6), SCI + saline (GFAP *n* = 6; IBA1 *n* = 6; CCL2 *n* = 6; CCR2 *n* = 7; CX3CL1 *n* = 6; CX3CR1 *n* = 6; CatS *n* = 6), SCI + GSE15 (GFAP *n* = 6; IBA1 *n* = 6; CCL2 *n* = 6; CCR2 *n* = 7; CX3CL1 *n* = 6; CX3CR1 *n* = 6; CatS *n* = 6), SCI + CE10 (GFAP *n* = 6; IBA1 *n* = 6; CCL2 *n* = 6; CCR2 *n* = 7; CX3CL1 *n* = 6; CX3CR1 *n* = 6; CatS *n* = 6). Figure S2 of Supporting Information file shows full-length blot images corresponding to the cropped Western blot presented in the figure.

In RVM, none of the analyzed markers followed a normal distribution (all *p* values < 0.01) according to the Shapiro–Wilk normality test. Further Kruskal–Wallis tests revealed significant differences in protein levels between groups (*p* < 0.01). Specifically, Mann–Whitney U tests indicated that all analyzed markers were increased in the SCI + saline group compared to the sham group (all *p* values < 0.01). Regarding polyphenolic treatments, both GSE15 and CE10 reduced GFAP, CX3CL1/CX3CR1, and CatS levels compared to those in the SCI + saline group (all *p* values < 0.05). Despite the reduction, the levels of these markers in animals treated with GSE15 and CE10 remained significantly higher than those observed in sham animals (all *p* values < 0.01), except for CX3CR1 in CE10-treated animals, which did not differ from the sham group (*p* = 0.065). Finally, levels of IBA1 and CCL2/CCR2 were significantly higher in both GSE15- and CE10-treated animals compared to non-treated SCI animals (all *p* values < 0.05), except for IBA1 in CE10-treated animals, which did not differ from the SCI + saline group (*p* > 0.09) (Figure 8).

### 2.4. Repeated GSE15 or CE10 Administration During the First, Third and Sixth Week Post-Injury Reduces Astrogliosis and Modulates CCL2/CCR2 and CX3CL1/CX3CR1 Signaling in the ACC and Amygdala at 10 Weeks Post-SCI

The anterior cingulate cortex (ACC) and amygdala (AMG) are recognized as crucial neural substrates involved in the affective component of pain [36]. Anatomically, the ACC shares robust connections with the AMG, which plays a role in processing mood, fear, emotional memory, and the emotional-affective aspect of pain [37]. Therefore, analysis of gliosis (marked by protein levels of GFAP and IBA1), protein levels of chemokines CCL2 and CX3CL1, their corresponding receptors CCR2 and CX3CR1, as well as cathepsin S (CatS) was conducted in these two supraspinal structures.

**Figure 9:**
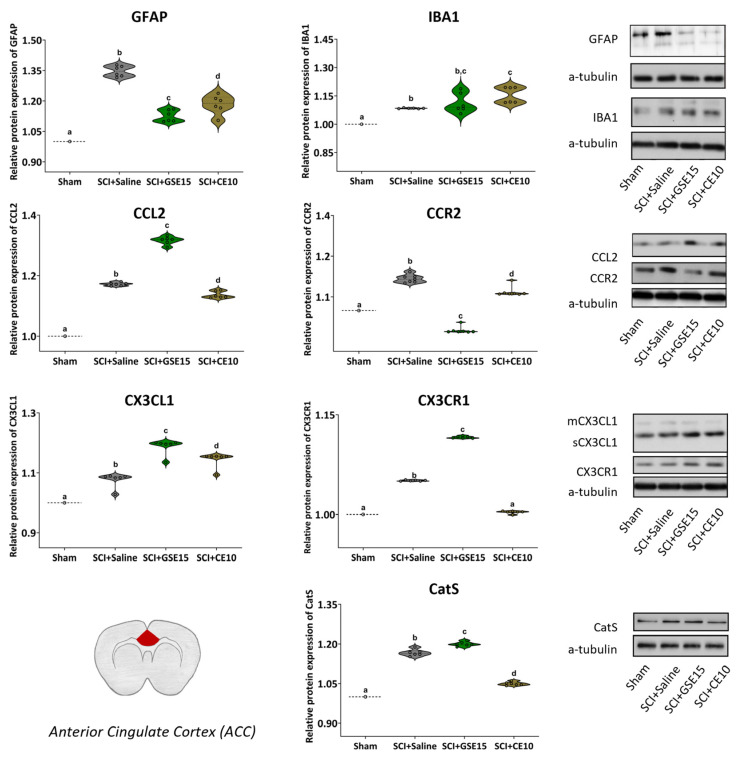
Expression of GFAP, IBA1, CCL2, CCR2, CX3CL1, CX3CR1 and CatS in ACC at the chronic phase of SCI-induced neuropathic pain after GSE15 and CE10 treatments in the first, third and sixth week post-injury. Protein expression was normalized to a-tubulin. Data are expressed as a relative percentage with respect to sham group (mean ± SEM). a–d: Groups not sharing a letter showed significant differences, *p* < 0.05. Experimental groups: sham (GFAP *n* = 6; IBA1 *n* = 6; CCL2 *n* = 6; CCR2 *n* = 7; CX3CL1 *n* = 6; CX3CR1 *n* = 6; CatS *n* = 6), SCI + saline (GFAP *n* = 6; IBA1 *n* = 6; CCL2 *n* = 6; CCR2 *n* = 7; CX3CL1 *n* = 6; CX3CR1 *n* = 6; CatS *n* = 6), SCI + GSE15 (GFAP *n* = 6; IBA1 *n* = 6; CCL2 *n* = 6; CCR2 *n* = 7; CX3CL1 *n* = 6; CX3CR1 *n* = 6; CatS *n* = 6), SCI + CE10 (GFAP *n* = 6; IBA1 *n* = 6; CCL2 *n* = 6; CCR2 *n* = 7; CX3CL1 *n* = 6; CX3CR1 *n* = 6; CatS *n* = 6). Figure S3 of Supporting Information file shows full-length blot images corresponding to the cropped Western blot presented in the figure.

In the ACC (Figure 9), none of the molecular markers followed a normal distribution based on the Shapiro–Wilk normality test (all *p* values < 0.05). Consequently, Kruskal–Wallis tests revealed significant differences between groups for all analyzed markers (all *p* values < 0.001). Notably, protein levels of GFAP, IBA1, CCL2/CCR2, CX3CL1/CX3CR1, and CatS were significantly increased in the SCI + saline group compared to the sham group (all *p* values < 0.01). Treatment with GSE15 reduced astrogliosis (GFAP) and the protein level of CCR2 in the ACC compared to SCI + saline animals (*p* < 0.01). Despite the reduction, the level of GFAP indicating astrogliosis remained significantly higher in GSE15-treated SCI animals than in sham animals (*p* < 0.01). In contrast, SCI animals treated with GSE15 exhibited similar (IBA1, *p* = 0.485) or higher protein levels (CCL2, CX3CL1, CX3CR1, and CatS; *p* < 0.01) compared to saline-treated SCI animals. CE10 treatment also reduced astrogliosis (GFAP) and the protein levels of CCL2/CCR2, CX3CR1, and CatS compared to SCI + saline animals (all *p* values < 0.01). However, the protein levels of all molecular markers remained significantly higher than those observed in sham animals (all *p* values < 0.01), except for CX3CR1, which did not differ from the sham group (*p* = 0.065) (Figure 9).

**Figure 10:**
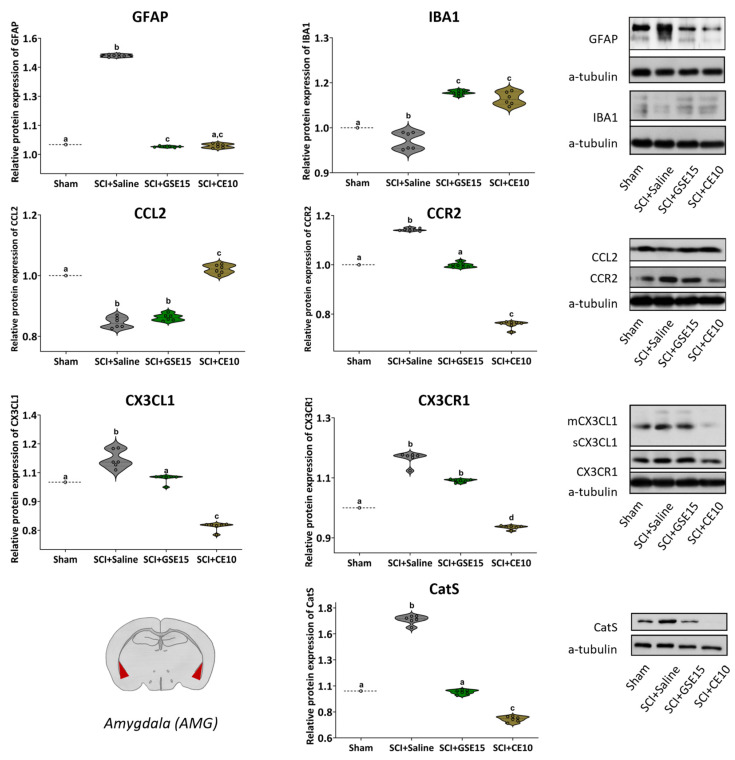
Expression of GFAP, IBA1, CCL2, CCR2, CX3CL1, CX3CR1 and CatS in AMG at the chronic phase of SCI-induced neuropathic pain after GSE15 and CE10 treatments in the first, third and sixth week post-injury. Protein expression was normalized to a-tubulin. Data are expressed as a relative percentage with respect to sham group (mean ± SEM). a–d: Groups not sharing a letter showed significant differences, *p* < 0.05. Experimental groups: sham (GFAP *n* = 6; IBA1 *n* = 6; CCL2 *n* = 6; CCR2 *n* = 7; CX3CL1 *n* = 6; CX3CR1 *n* = 6; CatS *n* = 6), SCI + saline (GFAP *n* = 6; IBA1 *n* = 6; CCL2 *n* = 6; CCR2 *n* = 7; CX3CL1 *n* = 6; CX3CR1 *n* = 6; CatS *n* = 6), SCI + GSE15 (GFAP *n* = 6; IBA1 *n* = 6; CCL2 *n* = 6; CCR2 *n* = 7; CX3CL1 *n* = 6; CX3CR1 *n* = 6; CatS *n* = 6), SCI + CE10 (GFAP *n* = 6; IBA1 *n* = 6; CCL2 *n* = 6; CCR2 *n* = 7; CX3CL1 *n* = 6; CX3CR1 *n* = 6; CatS *n* = 6). Figure S4 of Supporting Information file shows full-length blot images corresponding to the cropped Western blot presented in the figure.

In the AMG (Figure 10), none of the markers followed a normal distribution according to the Shapiro–Wilk normality test (*p* values < 0.05), with significant differences in protein levels between groups found using Kruskal–Wallis tests (*p* < 0.01). The protein levels of GFAP, CCR2, CX3CL1/CX3CR1, and CatS were significantly increased in the SCI +saline group compared to the sham group (all *p* values < 0.01), while IBA1 and CCL2 levels were lower in saline-treated SCI animals compared to sham animals (*p* values < 0.01). Both GSE15 and CE10 treatments significantly reduced the expression of GFAP, CCR2, CX3CL1, CX3CR1, and CatS compared to non-treated SCI animals (all *p* values < 0.01). GSE15 treatment reduced astrogliosis below sham levels (*p* < 0.01) and decreased protein levels of CCR2, CX3CL1, and CatS to levels similar to those in the sham group (*p* values > 0.05), although CX3CR1 level remained higher than that observed in the sham group (*p* < 0.01). CE10 treatment decreased protein levels of CCR2, CX3CL1, CX3CR1, and CatS to levels below those of the sham group (*p* values < 0.01) and reduced astrogliosis to levels similar to those observed in the sham group (*p* = 0.394) (Figure 10).

### 2.5. Repeated GSE15 or CE10 Administration During the First, Third and Sixth Week Post-Injury Does Not Trigger Weight Loss or Hepatotoxic or Nephrotoxic Effects in Spinal Cord-Injured Mice

In this study, adherence to animal welfare guidelines outlined by Morton and Griffiths (1985) [38], ensured the well-being of the animals under investigation, with no observed abnormalities in their general condition, coat, skin, vibrissae of nose, nasal secretions, or signs of autotomy or aggressiveness across all experimental groups of mice.

**Figure 11:**
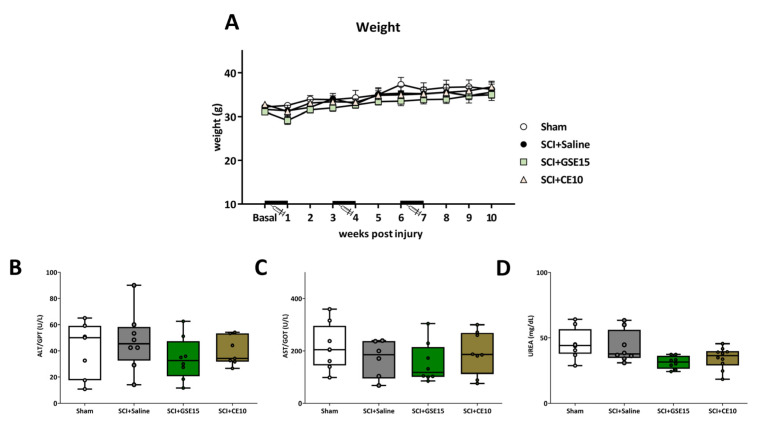
No systemic toxicity indicators after treatment. (**A**) Time-course assessment of mouse weights during the acute, intermediate and chronic phase of SCI-induced neuropathic pain after GSE15 and CE10 treatments in the first, third and sixth week post-injury. Each point and vertical line represents the mean ± SEM. Treatment administration weeks (basal to 1 wpi, 3 to 4 wpi and 6 to 7 wpi) are highlighted with a thick black line. Experimental groups: sham (*n* = 8), SCI + saline (*n* = 9), SCI + GSE15 (*n* = 9), SCI + CE10 (*n* = 10); Biomarker quantification of (**B**,**C**) hepatotoxicity and (**D**) nephrotoxicity in the serum of each experimental group at the end of experimental period. The results are represented as the mean ± SEM. Experimental groups: sham (ALT/GTP *n* = 7; AST/GOT *n* = 8; UREA *n* = 8), SCI + saline (ALT/GTP *n* = 8; AST/GOT *n* = 6; UREA *n* = 8), SCI + GSE15 (ALT/GTP *n* = 8; AST/GOT *n* = 8; UREA *n* = 8), SCI + CE10 (ALT/GTP *n* = 7; AST/GOT *n* = 8; UREA *n* = 10).

Various statistical analyses were employed to examine the impact of time and treatment on the animals’ weight. Despite the Kolmogorov–Smirnov normality test indicating a lack of normal distribution in the data (all *p* values < 0.05), the Friedman analysis revealed significant variation in the weight data throughout the experimental period (*p* < 0.001). However, subsequent Kruskal–Wallis tests indicatd no significant differences between groups at any experimental time points (all *p* values > 0.05), suggesting surgeries and treatments had no notable effects on weight (Figure 11A).

To evaluate potential hepatotoxic or nephrotoxic effects resulting from the administration of GSE15 and CE10, biomarkers of hepatotoxicity (ALT/GPT and AST/GOT) and nephrotoxicity (UREA) were quantified in the animals’ serum at the study’s conclusion. Following confirmation of normal distribution using the Kolmogorov–Smirnov test (all *p* values > 0.161), ANOVA analysis indicated no significant differences between experimental groups for any biomarkers: ALT/GPT (*p* = 0.543), AST/GOT (*p* = 0.612), and UREA (*p* = 0.179) (Figure 11B–D).

Overall, these findings indicate the absence of systemic toxicity linked to repeated administrations of either GSE (15 mg/kg) or CE (10 mg/kg; i.p.) throughout the first, third, and sixth weeks post-SCI. There were no observed hepatotoxic or nephrotoxic effects, as evidenced by the unchanged weight and appearance of the animals, along with unaltered biomarkers of hepatotoxicity and nephrotoxicity in their serum.

## 3. Discussion

The persistence of neuropathic pain following SCI is largely attributed to the absence of efficacious treatments [8,39,40], thereby increasing the likelihood of severe disabilities and susceptibility to emotional disorders [41,42]. While current approaches focus on polyphenols due to preclinical evidence of their antinociceptive properties [14,43], the dearth of effective interventions underscores the urgent need for novel pharmacological strategies to prevent chronicity in SCI-related neuropathic pain, given the rising number of patients entering chronic phases post-SCI.

Thus, this study was designed to explore a novel pharmacological approach targeting chronic neuropathic pain post-SCI by administering polyphenols throughout the pathophysiological phases of the injury, aiming to prevent the development of both reflexive and non-reflexive chronic pain responses. Specifically, building on previous findings indicating the efficacy of repeated GSE15 or CE10 polyphenolic extract administration in preventing the acute phase of neuropathic pain following SCI [17], the mouse experiments here presented involved administering GSE15 or CE10 repeatedly during the first, third, and sixth weeks post-injury to assess their potential in averting the development of chronic neuropathic pain. It is noteworthy that GSE and CE10 are thoroughly characterized natural polyphenolic extracts [17], comprising several major polyphenolic compounds potentially capable of eliciting synergistic effects that surpass those observed when polyphenols are administered individually [16,17]. In fact, considering insights from studies on refractory pathological pain, which indicate that single-drug administration may not exert sufficient efficacy [44], the rationale behind the effectiveness of these extracts likely lies in the utilization of a blend of diverse polyphenols rather than individual ones.

As mentioned before, the doses of 15 mg/kg (GSE15) and 10 mg/kg (CE10) were chosen based on our prior preclinical research [17,22], which demonstrated robust antinociceptive and anti-inflammatory effects. To our knowledge, no human clinical trial or published report has focused exclusively on using polyphenols to alleviate SCI-induced neuropathic pain. Nevertheless, meta-analyses of polyphenol interventions for pain conditions such as osteoarthritis and rheumatic diseases [45,46] report dosage ranges that, when converted into mg/kg, align with ours. For example, daily human intakes ranging from 42 mg to 1585 mg of polyphenol-rich sources [46] (approximately 0.6–22.6 mg/kg/day for a 70 kg adult) are comparable to our 10–15 mg/kg range. Likewise, those analyses highlight that polyphenol-rich extracts remain effective at dose levels matching ours [45] once human-to-rodent conversions are considered. Consequently, our chosen doses fit well within the lower-to-mid range reported in the literature and support both the safety and efficacy of these doses in preventing or alleviating SCI-induced outcomes.

While previous studies have indicated comparable levels of neuropathic pain between male and female patients following spinal cord injury (SCI) [47,48], investigations into pathological pain post-SCI have predominantly focused on young male rodents [49]. However, epidemiological evidence suggests that females exhibit a higher prevalence of chronic pain and are more susceptible to developing pain-related comorbidities, including emotional disorders [50,51]. This underscores the necessity of enhancing the representation of female subjects in experimental designs. To maintain consistency, female CD1 mice were utilized, given their widespread use in pharmacological assessments [52,53] and their low levels of anxiety, which are crucial for evaluating pain behaviors in animal models [54]. Specifically, our study employed a suitable female mouse model, ensuring alignment with previous experimental findings [18,21,55,56]. Regarding the type of SCI, it is important to note that severe injuries can lead to significant locomotor disturbances, potentially complicating the evaluation of neuropathic pain and hindering behavioral assessments requiring free movement. Therefore, a mild spinal cord contusion was induced following established procedures [21], to enable the development of chronic central neuropathic pain in mice without inducing locomotor paralysis.

The primary outcomes of the present investigation revealed that both polyphenolic treatments successfully prevented the onset of reflexive pain responses for up to 10 weeks following SCI, spanning into the chronic phase of the condition. Until now, the potential effects of polyphenols on preventing chronic central neuropathic pain have remained elusive. To our understanding, these findings provide compelling evidence for these promising effects. These experimental results indicate a gradual reduction in reflexive neuropathic pain responses in SCI animals, extending into the late-chronic phase, as recently observed in CD1 SCI chronic mice [21]. It remains challenging to determine whether these heightened withdrawal thresholds correspond to diminished hypersensitivity or other factors like habituation to testing protocols. While withdrawal from thermal or mechanical force represents an evoked response driven by spinal cord circuitry [57], conscious decision-making regarding pain or discomfort is likely absent in these reflexive responses [58]. Therefore, relying solely on evaluating evoked-reflexive pain responses may not provide a comprehensive assessment of pain during the chronic phase of SCI. In line with this understanding, our recent findings have demonstrated an increase in non-reflexive pain responses up to the chronic phase of SCI, while reflexive responses were decreasing [21]. It is important, then, to determine whether polyphenolic treatment could also exert preventative effects on chronic non-reflexive pain responses.

Accordingly, in addition to reflexive pain responses, we assessed social, anxiety-, and depressive-like behaviors as non-reflexive pain responses. Specifically, the light and dark box, open field, forced swim, and social interaction tests were conducted during the chronic phase of the injury, precisely between the ninth and tenth weeks post-SCI. Overall, our investigation into the modulation of the non-reflexive effects of SCI on female CD1 mice revealed notable outcomes. Specifically, we observed that administration of the polyphenolic extracts during the first, third, and sixth weeks after SCI effectively prevented the development of chronic non-reflexive pain responses, such as anxiety and depressive-like behaviors, as well as chronic sociability alterations. These experimental results are particularly noteworthy given that, in addition to experiencing pain, individuals with SCI often manifest significant symptoms of emotional mood disorders [59,60], surpassing the global prevalence of depression and anxiety by approximately fivefold [61,62]. Moreover, our findings underscore the heightened significance for females, since, as mentioned before, they are more vulnerable to experiencing neuropsychological distress following SCI [63,64].

In humans, SCI frequently results in immune dysregulation, triggering an early systemic inflammatory response characterized by elevated levels of circulating proinflammatory mediators [65,66,67]. Furthermore, research suggests that these proinflammatory mediators may interact with specific brain regions, potentially influencing behavioral changes [68]. Additionally, it has been suggested that maintaining a chronic inflammatory state contributes to the development of psychiatric disorders following SCI [62,69,70,71]. Moreover, recent findings underscore the involvement of homeostatic glia in the regulation of neuronal activity, synaptic interactions, tuning of neural circuits, and modulation of behaviors. Given this body of research, it is plausible to hypothesize that polyphenolic extracts could modulate these inflammatory factors, potentially mitigating the development of SCI-induced non-reflexive pain responses. In this line, our recent study demonstrated that in the female chronic-SCI mouse model, transient reflexive responses were observed during the acute phase of SCI, accompanied by a temporary overexpression of cytokines in the spinal cord. Conversely, increased non-reflexive pain responses were noted during the chronic phase, coinciding with cytokine overexpression in supraspinal structures [21]. In turn, our recent research has shown that the administration of either CE or GSE extracts modulates reflexive pain responses by regulating gliosis in both spinal cord and supraspinal structures involved in pain processing. Additionally, these extracts prevent the upregulation of chemokines and their receptors associated with the development of pathological pain, such as CX3CL1/CX3CR1 [17]. Thus, considering these findings, molecular analyses were scheduled to gain mechanistic insights into the beneficial effects of polyphenolic extracts in preventing both reflexive and non-reflexive chronic pain responses observed in our present experiments. Specifically, our focus was on investigating gliosis and neuroimmune crosstalk processes involving chemokine pathways CX3CL1/CX3CR1 and CCL2/CCR2 in supraspinal structures associated with both pain modulation and pain affective responses. Consequently, our molecular outcomes showed that both GSE and CE treatments modulated astrogliosis, as well as CCL2/CCR2 and CX3CL1/CX3CR1 signaling in the ACC, AMG, dlPAG, vlPAG, and RVM, and microgliosis in the vlPAG at 10 weeks post-SCI.

Regarding the RVM and PAG, our findings indicated that the repeated administration of polyphenolic extracts reduced astrogliosis and modulated CCL2/CCR2 and CX3CL1/CX3CR1 signaling pathways at the chronic SCI phase. The RVM and PAG are known to be central components of the internal modulatory system, influencing spinal cord sensory processing [30], although their precise mechanisms remain unclear. The vlPAG has been linked to social behaviors, while the dlPAG is more involved in pain responses and fear [72,73]. Our study revealed the activation of glial cells in both the vlPAG and dlPAG following chronic SCI, indicating their potential contribution to increased pain sensitivity and behavioral alterations. Concurrently, considering that glial activation in the RVM post-injury corresponds with its role in promoting heightened pain sensitivity in various pathological conditions [74,75,76], the glial modulation in the RVM after treatments may also account for the beneficial effects of GSE and CE on mitigating the development of chronic pathological pain post-SCI.

Alongside gliosis, our current investigation unveiled the elevated levels of chemokines CCL2 and CX3CL1, their receptors CCR2 and CX3CR1, and CatS, the protease responsible for soluble CX3CL1 secretion, in the PAG and RVM of mice with SCI. It is unsurprising to observe such an increase in these markers, given that activated glial cells are known for enhancing the synthesis and release of various chemokines implicated in signaling between neurons and glia, as well as in the modulation of nociceptive transmission [77,78,79,80]. Notably, repeated administration of polyphenolic extracts prevented this increase in chemokines and their receptors, thereby averting the development of chronic pain responses and suggesting this modulation as a primary potential mechanism. To the best of our knowledge, no prior studies have demonstrated an elevation in these chemokines and their receptors within both the PAG and RVM during the chronic phase post-SCI, apart from one investigation indicating increased CCL2 expression in the PAG following severe SCI with prolonged neuropathic pain behavior [81]. In this context, intrarapheal injections of CCL2 induced dose-dependent hyperalgesia [82]. Furthermore, intra-PAG administration of CX3CL1 prior to mu, delta, and kappa opioid agonist administration has been demonstrated to significantly attenuate the antinociceptive effects of opioids, indicating a critical role of chemokines in the PAG for opioid treatment of chronic pain [83]. Therefore, our findings, corroborated by the aforementioned literature, suggest that the upregulation of the CCL2/CX3CL1 chemokine network and the endogenous opioid system within the PAG-RVM axis, known to interact with each other, may serve as pivotal components in the cascades underlying the development of central neuropathic pain after SCI, and the polyphenolic repeated administration would be preventing these pathways’ activation.

Similar results were obtained in the supraspinal structures associated with emotional pain responses: amygdala (AMG) and anterior cingulate cortex (ACC). Our results revealed an increase in glial reactivity, as well as the expression of chemokines CCL2/CX3CL1 and their receptors CCR2/CX3CR1, and CatS in the ACC. In parallel, astrogliosis and overexpression of CX3CL1/CX3CR1, CatS, and CCR2 were observed in the AMG of animals with SCI at 10 weeks post-injury. The observed changes in supraspinal structures were accompanied by depressive- and anxiety-like behaviors, as well as social behavior disturbances in the chronic phase of SCI. The repeated administration of polyphenolic extracts prevented these cellular and molecular processes associated with the development of non-reflexive pain responses.

With regard to the AMG, it is recognized for its involvement in the processing mood, fear, emotional memory, and the emotional-affective aspect of pain [37]. Several neuroimaging studies have observed pain-related alterations in the AMG across various pain conditions [84,85]. Specifically, the central AMG nucleus (CeA) has been designated the “nociceptive amygdala” [86,87], and an augmented number and excitability of CeA neurons have been associated with depressive-like behavior in neuropathic pain [88]. Depending on environmental factors and affective states, the AMG appears to exert both facilitatory and inhibitory effects on pain behavior and nociceptive processing at different levels of the pain neuraxis [86]. Besides the AMG, the ACC has also been implicated in both anxiety and pain in both human and animal studies [89,90]. Anatomically, it has long been known that the ACC exhibits robust connections with other cortical and subcortical regions, including the AMG, mediodorsal thalamic nuclei, hypothalamus, hippocampal formation, and various cortical areas, all of which are involved in emotion, behavioral control, and pain processing [80,91,92,93]. These findings provide a structural basis for the contribution of the ACC and AMG to pain affect.

The alterations in the ACC associated with neuropathic pain, particularly gliosis and CX3CL1/CX3CR1 expression, were previously observed during the acute phase of the SCI [21]. According to the present findings, these alterations continue and exert a pivotal role in the affective component of neuropathic pain during the chronic phase of SCI. On the other hand, to our knowledge, there are currently no studies describing the expression pattern of CCL2/CCR2 in the ACC of chronic SCI-induced neuropathic pain models. Regarding changes in the AMG, a significant increase in GFAP was detected in the AMG of another experimental model of neuropathic pain based on a spared nerve injury (SNI) of the sciatic nerve [94]. While upregulation of CCR2 in the AMG associated with neuropathic pain has not previously been reported, an overexpression of CCL2 in the CeA of mice with partial sciatic nerve ligation (PSNL) that exhibited anxiety-like behavior and hypersensitivity to mechanical stimuli has been demonstrated [95]. Regarding CX3CL1/CX3CR1 signaling, Cho et al. recently demonstrated increased CX3CR1 expression in both the hippocampus and AMG in a rat model of postoperative pain [96]. These findings are consistent with our results and collectively support the notion that non-reflexive pain responses, such as anxiety and depression, are mediated by changes in the ACC [97,98,99], and AMG nuclei [100].

It is worth mentioning that polyphenolic extracts from grape stalk (GSE) and coffee (CE) differ from standard NSAIDs and corticosteroids by modulating multiple neuroimmune pathways [101]. They not only inhibit inflammation but also reduce supraspinal gliosis through CCL2/CCR2 and CX3CL1/CX3CR1 signaling [102]. Unlike conventional anti-inflammatories, which primarily target COX enzymes, these extracts address oxidative stress, protect mitochondrial function, and confer significant neuroprotection [103,104]. Our study showed that repeated GSE and CE administration prevented mechanical allodynia and thermal hyperalgesia for up to 10 weeks post-SCI, surpassing the typical duration of standard anti-inflammatory drugs. By crossing the blood–brain barrier, polyphenols likely modulate central pain processing [105] and exhibit fewer adverse effects than prolonged NSAID or steroid use [106,107]. Critically, their ability to prevent the transition from acute to chronic pain highlights their disease-modifying potential.

In summary, from the repeated administration of polyphenolic extracts assessed in the present study, it was observed that both GSE15 and CE10 modulated astrogliosis, CCL2/CCR2, and CX3CL1/CX3CR1 signaling in the ACC, AMG, dlPAG and vlPAG, as well as the RVM, alongside microgliosis in the vlPAG at 10 weeks post-SCI. The polyphenols present in these extracts exhibit the ability to prevent the development of pain behaviors not only during the acute phase of the SCI but also up to the chronic phase, particularly when administered repeatedly over time, coinciding with the onset of the acute, intermediate, and chronic phases. Consequently, these findings, at both the molecular and behavioral levels, hold significant implications, paving the way for preventive interventions targeting the emotional disorders associated with central neuropathic pain following SCI.

Finally, while our findings indicate that repeated polyphenol administration may prevent SCI-induced neuropathic pain with minimal apparent side effects, key challenges remain for clinical translation. First, large-scale, long-term trials are needed to evaluate the safety profiles, potential drug interactions, and bioavailability under real-world conditions. Second, as previously noted, only about one-third of patients respond to existing neuropathic pain treatments when compared with placebo [40], often with adverse effects. Moreover, single-drug therapies may be insufficient for refractory pathological pain [44], suggesting that polyphenol-rich interventions could fill a critical gap if shown to be both efficacious and tolerable over time. Finally, standardizing doses and identifying the patient subpopulations most likely to benefit require further investigation. Overall, these promising experimental outcomes must be corroborated by robust clinical studies to confirm the long-term viability of polyphenolic extracts relative to existing therapies.

## 4. Materials and Methods

### 4.1. Experimental Design

This study aimed to assess the preventive effects of two polyphenolic extracts, grape stalk extract (GSE) and coffee extract (CE), on the development of chronic pain following SCI in mice. Female CD1 mice underwent mild spinal cord contusion and received daily treatments of GSE or CE during the first, third, and sixth weeks post-surgery. Reflexive pain responses, including thermal hyperalgesia (assessed via the Hargreaves test) and mechanical allodynia (evaluated using von Frey filaments), along with locomotor functional recovery (measured by the Basso mouse scale, BMS), were monitored weekly. Non-reflexive pain responses were evaluated using open field, dark and light box, forced swim, and social interaction tests during the final week of the experimental period. Furthermore, the safety of GSE and CE treatments was assessed by analyzing serum levels of hepatotoxicity and nephrotoxicity biomarkers at the end of the experimental period. Finally, molecular studies to assess neuroimmune crosstalk modulation were conducted by investigating gliosis and the expression of CCL2/CCR2 and CX3CL1/CX3CR1 in supraspinal structures implicated in sensory-discriminative and affective-motivational aspects of pain, including the anterior cingulate cortex (ACC), amygdala, dorsal and ventral periaqueductal gray (PAG), and rostroventromedial medulla (RVM).

### 4.2. Animals

Adult female ICR CD1 mice weighing between 20 and 25 g were sourced from Janvier Laboratories (Le Genest-Saint-Isle, France). Experimental groups were limited to 9–10 mice each to minimize the number of animals used. The required sample size for functional evaluation was determined using GRANMO (Version 7.12 April 2012) and the University of Boston spreadsheet (Sample Size Calculations (IACUC); Boston University), adhering to ethical guidelines established by the Animal Ethics Committee. Mice were housed in standard plexiglass cages measuring 28 × 28 × 15 cm, provided ad libitum access to food and water, and maintained under a 12:12 h light/dark cycle at a temperature of 21 ± 1 °C with 40–60% humidity. Cage bedding was changed twice weekly. Prior to any functional or surgical procedures, mice were allowed to acclimate to the facility rooms for at least 1 h, all of which were performed during the light cycle. Regular testing of sentinel mice ensured pathogen-free facilities throughout the experimental period.

All experimental procedures and animal care adhered to the ARRIVE 2.0 guidelines and ethical principles outlined by the I.A.S.P. for pain assessment in conscious animals [108], as mandated by the European Parliament and Council Directive of 22 September 2010 (2010/63/EU). The study protocol received approval from the Animal Ethics Committee of the University of Barcelona and the Generalitat de Catalunya, Government of Catalonia (num. 9918-P3). Every effort was made to minimize animal discomfort and reduce the number of animals used while ensuring consistent procedure outcomes.

### 4.3. Surgical Procedure and Pharmacological Treatment with Polyphenolic Extracts

Mild spinal cord contusion was performed following previously described protocols [21,55,56]. Briefly, animals were anesthetized with sodium pentobarbital (50 mg/kg, i.p.) and positioned prone on a heating pad to maintain stable body temperature. Following back shaving and disinfection with povidone iodide, the T8–T9 thoracic spinal cord segments were exposed through dorsal laminectomy, and a weight of two grams was dropped from a height of 25 mm onto a metallic stage positioned over the exposed spinal cord. Subsequently, the wound was closed and disinfected with povidone. Animals were then administered 0.5 mL of saline solution to address potential blood volume deficits and were allowed to recover in warmed cages with access to food and water. In sham animals, used as controls, the T8–T9 thoracic spinal cord segments were exposed via dorsal laminectomy but they were not subjected to contusion.

### 4.4. Treatment with Polyphenolic Extracts

The study utilized two fully characterized extracts sourced from different natural origins [17]. The grape stalk extract (GSE) was derived from the leftover grape stalks of Cabernet Sauvignon and Merlot varieties, sourced from winemaking processes in Catalonia’s L’Empordà region, graciously provided by Celler Roig Parals winery in Mollet de Peralada, Catalonia. Plant collection adhered to national guidelines and regulations. The coffee extract (CE) was obtained from commercially available decaffeinated ground roasted coffee, a blend of Robusta (pure *Coffea canephora*) and Arabica (pure *Coffea arabica*) beans roasted to a dark-medium level (7 out of 10). GSE particles were obtained by chopping grape stalks and sieving them to achieve a size of 0.5–1 mm. CE was prepared using processed coffee powder. Extracts were made by mixing 3 g of either material with 50 mL of saline solution, refluxing, and stirring at 100 °C for 2 h. The resulting solutions were filtered through progressively smaller pore size filters, with the final filtration using a 0.22 micrometer pore diameter filter, followed by sterilization before use [17]. Multiple batches of both GSE and CE were acquired for experimental use, with their total polyphenolic content through Folin–Ciocalteu assay. GSE exhibited an average total polyphenolic content of approximately 1100 mg GAE/L, while CE averaged around 2200 mg GAE/L. HPLC analysis revealed gallic acid, protocatechuic acid, and catechin as major polyphenols in GSE, and chlorogenic acid, neochlorogenic acid, and cryptochlorogenic acid as primary polyphenols in CE, with others identified at lower concentrations [17]. Major polyphenol concentrations for both extracts are shown in Table 1.

The extracts were administered intraperitoneally at doses of 15 mg/kg for GSE and 10 mg/kg for CE throughout the acute, intermediate, and chronic phases of SCI. Although humans may require up to six months to reach the chronic stage of SCI [2], the corresponding timeline in murine models is notably shorter: the immediate and acute phases typically last for approximately 2–3 weeks [26], followed by an intermediate phase spanning 3–6 weeks [27]. Thereafter, the chronic phase usually commences around 6–8 weeks post-injury [28]. On this basis, the extracts were administered at the beginning of each SCI phase, corresponding to the first, third, and sixth weeks post-surgery. These doses were chosen because they have previously demonstrated efficacy in both preventing the onset of acute pathological pain in mice [16,17], or alleviating it once present [22].

### 4.5. Locomotor Activity Evaluation

It was assessed using a circular open field (70 cm diameter × 24 cm wall height), allowing each animal 5 min of unrestricted movement. Two independent observers evaluated hindlimb movements and rated locomotor function based on the Basso mouse scale for locomotion (BMS) [109]. The final score for each animal was the average of both examiners’ scores. BMS scores range from 0 (no hindlimb movement) to 9 (normal, coordinated gait). The aim of this assessment is to ascertain the animals’ locomotion status post-spinal cord injury, ensuring all exhibit BMS scores higher than 5–6, indicating retained mobility and hind leg voluntary movement.

### 4.6. Reflexive Pain Response Assessment

Mechanical allodynia. This was assessed by determining 50% withdrawal thresholds using von Frey monofilaments with a bending force range of 0.04–2 g, following the up–down paradigm [17,21]. Animals were housed in plastic tube test chambers with metal mesh floors, allowing access to their hind paws’ plantar surfaces. After approximately 1 h of behavioral accommodation, von Frey monofilaments were applied perpendicular to the plantar surface with enough force to cause slight buckling. Filaments were sequentially applied, starting with a 0.4 g filament, adjusting the force based on the mouse’s response. Responses included paw withdrawal, shaking, or licking. This up–down procedure was limited to four assessments after the initial response. Each filament was applied for 2 s with interstimulus intervals of 5–10 s. Both hind paws were evaluated due to bilateral injury in the SCI model, precluding the use of the contralateral paw as a natural control. The 50% paw withdrawal threshold was calculated using the Dixon formula [110].

Thermal hyperalgesia. This was evaluated by assessing hind paw withdrawal latency in response to a thermal stimulus using the Hargreaves method in a plantar test algesimeter (#37370; Ugo Basile, Gemonio, Comerio, Italy) [111]. Mice were placed in plastic test enclosures with an elevated glass floor for approximately 1 h until major grooming activities ceased. A projection lamp (100 W) was then focused onto the plantar surface of the hind paw with a 30 s time limit to prevent skin damage. Withdrawal latency was automatically recorded using infrared detectors directed at the paw’s plantar surface. The mean withdrawal latencies for both hind paws were calculated from three separate trials conducted at 5 min intervals [17,21].

### 4.7. Non-Reflexive Pain Responses Assessments

Open field test. This utilizes innate rodent behaviors upon encountering a new environment and is widely employed for assessing anxiety-like behavior in mice [29,112]. Each mouse was positioned at the edge of a white Plexiglass enclosure (50 × 50 × 45 cm) under low-lighting conditions (15–20 lux) and recorded from above for 10 min as it explored the arena freely. Movement behaviors were analyzed, including overall activity, time spent in peripheral, middle, and central zones, entries into zones, latency to entry, and zone transitions, using Panlab Smart video tracking software (SMART V3.0.06; Harvard apparatus, Barcelona, Spain).

Light and dark box test. This is frequently employed to evaluate anxiolytic-like behavior in mice [113]. The test setup comprised a rectangular box divided into two compartments: a dark chamber (16 × 25 × 24 cm) occupying one-third of the space, and a light chamber (25 × 25 × 24 cm) occupying two-thirds, connected by a door (7 × 10 cm) at floor level. The dark chamber, covered with a roof, maintained darkness (0–5 lux), while the light chamber was illuminated dimly (20–25 lux), with an intermediate zone around the door. Animals were individually placed in the dark chamber, facing away from the door, and allowed to explore for 10 min while recorded by a video camera overhead. Panlab Smart video tracking software (SMART V3.0.06; Harvard apparatus, Spain) measured time spent in each chamber, overall activity, entries into the light chamber, and crossings between chambers. Increased time and entries into the light chamber typically correlate with reduced anxiety-like behavior [114].

Forced swimming test. This widely used depression test for mice, introduced by Porsolt et al., 1977 [115], capitalizes on their innate escape-oriented mobility when placed in a transparent cylinder filled with water. This behavior, marked by agitation for escape and immobility for energy conservation, is indicative of depressive states. Post-antidepressant treatment, mice exhibit increased struggling and reduced immobility, reflecting improved mood. Mice were placed individually in glass cylinders (40 cm height, 15 cm diameter) filled with water (25 ± 1 °C) to a depth of 30 cm, with a test duration of 6 min. The Panlab Smart video tracking software (SMART V3.0.06; Harvard apparatus, Spain) analyzed global activity, activity levels (percentage of immobility, low and high activity), and immobility instances.

Social interaction test. This was conducted in cages featuring two grid enclosures positioned opposite each other within the arena, following Crawley’s social interaction test [116]. The experimental test consisted of three trials: habituation (adaptation), social affiliation aspect of the test (session I) and social novelty/preference section of the test (session II). For the habituation, subject mouse was placed at the middle of the apparatus and allowed to acclimate for 5 min. For session I, one of the control mice (“Stranger mouse 1”) was placed inside a grid enclosure located in one of the side areas. Then, subject mouse was placed at the middle of the apparatus and allowed to freely explore the areas for 10 min while video camera recorded from above. The placement of stranger mouse 1 in the left or right side of the chamber was systematically altered between trials. For session II, a second control mouse (“Stranger mouse 2”) was placed inside an identical grid enclosure located in the opposite side area (that had been empty during session I). Then, subject mouse was placed in the middle of the apparatus and allowed to freely explore the areas for 10 min while video camera recorded from above.

Four measures were assessed over a 10 min period: direct interaction (comprising nose-to-nose and nose-to-tail contacts); latency to first direct interaction, which denotes the time until the tested mouse initiated direct interaction with the novel mouse, excluding unspecific contacts; preference time in stranger mouse area, which is the net time that the tested mouse spent in the stranger mouse 1 or 2 side areas; and entries into the stranger mouse area, referring to the number of times that a tested mouse entered the stranger mouse 1 or 2 side areas.

### 4.8. Biological Sample Collection

At the end of the experimental period, the animals were deeply anesthetized using sodium pentobarbital (90 mg/kg; i.p.), ensuring complete sedation. Samples of serum, and supraspinal structures were then meticulously collected. Upon reaching the anesthesia-induced plane characterized by the absence of foot reflex and abdominal breathing, the animals were positioned in a supine decubitus posture, and blood was extracted via an intracardiac needle insertion. The extracted blood underwent centrifugation at 4000 rpm for 15 min to isolate the serum, which was promptly frozen using dry ice and stored at −80 °C for subsequent analysis. The entire brain was excised from each mouse’s cranium and preserved at −80 °C for further examination.

### 4.9. Western Blotting Analysis

To acquire the PAG region, a 2 mm thick coronal slice of the mesencephalon was meticulously excised, spanning from the central portion of the superior colliculi to the upper extent of the inferior colliculi, aligning with PAG coordinates of 6.72 mm to 8.04 mm from the Bregma as per Paxinos and Watson (1997) [117]. Following this, radial segments corresponding approximately to ipsilateral and contralateral dorsolateral PAG (dlPAG) and ventrolateral PAG (vlPAG) were dissected under a stereomicroscope, adhering to anatomical criteria outlined by Keay and Bandler (2001) [118]. To isolate the rostral ventromedial medulla (RVM), a 2 mm coronal slice of the medulla was made, located 1 mm posterior from the inferior colliculi edge, approximately 1 mm from the interaural line, corresponding to RVM coordinates between −10.8 mm and −11.4 mm from the bregma [119]. Subsequently, a tissue triangle was dissected under a stereomicroscope to encompass the RVM area, including the nucleus raphe magnus, gigantocellularis, and gigantocelularis pars alpha. For the ACC, a 2 mm thick coronal slice of the prefrontal cortex was precisely removed, positioned between 2.2 mm and 4.2 mm from the Bregma according to Paxinos and Franklin (2013) [119]. Lastly, for the amygdala (AMG), a 1.5 mm thick coronal section was meticulously prepared at coordinates between −1.5 and −3.0 mm from the Bregma [119], followed by dissection of a triangular segment containing amygdalar nuclei. Afterwards, the ACC, AMG, vlPAG, dlPAG, and RVM tissue samples of individual animals were homogenized in RIPA buffer (Abcam, Cambridge, UK) containing protease inhibitors (LaRoche, Basel, Switzerland), and were then centrifuged at 15,000× *g* at 4 °C for 20 min. Protein concentration from the tissue supernatant was measured by Nanodrop ND-1000 (Thermo Scientific, Waltham, MA, USA) and normalized to the same levels. Each sample, containing 50 µg of protein, was separated by SDS-polyacrylamide gel electrophoresis, and transferred onto nitrocellulose membranes. The membranes were then blocked with 1% BSA in PBST (3.2 mM Na_2_HPO_4_, 0.5 mM KH_2_PO_4_, 1.3 mM KCl, 135 mM NaCl, 0.05% Tween 20, pH 7.4) for 1 h at room temperature and incubated with the following primary antibodies overnight at 4 °C: Rabbit Anti-IBA1 (1:100, FUJIFILM Wako Chemicals, Richmond, VA, USA), Rabbit Anti-GFAP (1:10,000, Abcam), Rabbit Anti-MCP1 (1:1000, ab25124, Abcam), Rabbit Anti-CCR2 (1:1000, ab203128, Abcam), Rabbit Anti-CX3CL1 (1:2000, Abcam), Rabbit Anti-CX3CR1 (1:2000, Abcam), and Rabbit Anti-CatS (1:1000, Abcam). Mouse Anti-α-tubulin antibody (1:1000, Cell Signaling, Danvers, MA, USA) was used as a loading control. Blots were washed in PBST and incubated with peroxidase-conjugated anti-mouse or anti-rabbit secondary antibodies (Sigma, 1:1000, St. Louis, MO, USA) at room temperature for 1 h. Protein bands were visualized using the ECL detection kit (Amersham, Chicago, IL, USA) on an LAS-3000 chemiluminometer reader (Fuji, Tokyo, Japan) and analyzed using densitometry image software.

### 4.10. Biochemical Analysis of Hepato- and Nephrotoxicity

Serum samples underwent analysis to investigate potential hepatotoxicity and nephrotoxicity induced by the various polyphenols found in the extracts. For this purpose, the Alanine Aminotransferase (ALT/GPT) kit (COD 11533, BioSystems, Barcelona, Spain), Aspartate Aminotransferase (AST/GOT) kit (COD 11531, BioSystems), and Urea-BUN color kit (COD 11536, BioSystems) were employed. Additionally, the analysis utilized a thermostatic water bath set at 37 °C and a spectrophotometer (ZUZI, model 4211/20) capable of measuring at 600 nm (UREA) or 340 nm (AST/ALT) [17,22].

### 4.11. Statistical Analysis

All functional, histological, and biochemical analyses were conducted in a blinded manner, with each mouse assigned a code for identification. Results are presented as mean ± standard deviation of the mean (SEM). Prior to further analysis, the normal distribution of data was assessed using the Kolmogorov–Smirnov test. Parametric or non-parametric statistical methods were then applied accordingly. Data exhibiting normal distribution underwent analysis using repeated measures MANOVA (Wilks’ criterion) and analysis of variance (ANOVA), followed by Duncan’s test where applicable. For data not conforming to a normal distribution, the Friedman statistic test for non-parametric repeated measures and Kruskal–Wallis test followed by Mann–Whitney U test were employed. The significance level (α) for all statistical analyses was set at 0.05, and the analyses were conducted using SPSS 25.0 for Windows.

## 5. Conclusions

In conclusion, we have demonstrated that a mixture of polyphenols present in natural extracts may be a suitable pharmacological strategy to prevent the development of SCI-induced neuropathic pain up to the chronic phase by modulating not only the reflexive pain responses (more related to the sensory-discriminative dimension of pain) but also the non-reflexive pain responses (included in the affective-motivational dimension of pain). These compounds not only exerted modulatory effects at the site of injury by modulating gliosis and the expression of markers related to central sensitization but could also play pivotal roles on supraspinal structures closely related to the expression and modulation of central neuropathic pain. Hence, to our knowledge this is the first study showing that both GSE and CE may be suitable pharmacological treatments to modulate both reflexive and non-reflexive pain response development up to the SCI chronic phase, without displaying systemic toxicity and they may be also useful for translation into clinics for novel therapeutic strategies.

## Figures and Tables

**Table 1:** Major polyphenols in GSE and CE quantified by HPLC-UV-ESI-TOFMS. Quantification of each polyphenol identified was performed in triplicate and all values are presented as mean ± SEM (*n* = 3). The molecular structures of major compounds are also presented.

**Grape Stalk Extract—GSE**			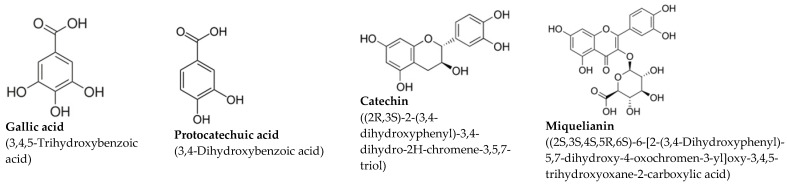
**Polyphenol**	[mg/L]	SEM	
Gallic acid	39.55	1.3	
Protocatechuic acid	30.5	0.9	
Catechin	29.2	0.7	
Miquelianin	18.12	1.6	
Caftaric acid	11.5	0.7	
**Coffee Extract—CE**			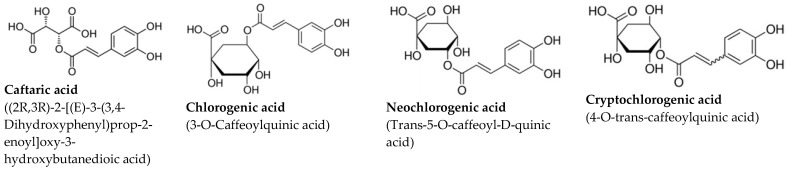
Polyphenol	[mg/L]	SEM	
Chlorogenic acid	339.2	12.9	
Neochlorogenic acid	308.1	14.3	
Cryptochlorogenic acid	307.6	18.2	

## Data Availability

The original contributions presented in the study are included in the article, further inquiries can be directed to the corresponding authors.

## Supplementary Materials

The following supporting information can be downloaded at: https://www.mdpi.com/article/10.3390/ijms26073325/s1.

## Abbreviations

The following abbreviations are used in this manuscript:

ACC	Anterior cingulate cortex
ALT	Alanine aminotransferase
AMG	Amygdala
AST	Aspartate aminotransferase
BMS	Basso mouse scale
CatS	Cathepsin S
CCL2	Chemokine (C-C motif) ligand 2
CCR2	Chemokine (C-C motif) ligand 2 receptor
CE	Coffee extract
CNP	Central neuropathic pain
CX3CL1	Chemokine (C-X3-C motif) ligand 1
CX3CR1	Chemokine (C-X3-C motif) ligand 1 receptor
dlPAG	Dorsolateral periaqueductal gray
GFAP	Glial fibrillary acidic protein
GSE	Grape stalk extract
RVM	Rostroventromedial medulla
SCI	Spinal cord injury
vlPAG	Ventrolateral periaqueductal gray
wpi	Weeks post injury
