# Supplementary material for: Repeated Administrations of Polyphenolic Extracts Prevent Chronic Reflexive and Non-Reflexive Neuropathic Pain Responses by Modulating Gliosis and CCL2-CCR2/CX3CL1-CX3CR1 Signaling in Spinal Cord-Injured Female Mice
Source: Int J Mol Sci. 2025 Apr 2;26(7):3325. doi: 10.3390/ijms26073325 (PMC11989601; doi:10.3390/ijms26073325)

Supplementary Materials

Repeated Administrations of Polyphenolic Extracts Prevent Chronic Reflexive and Non-Reflexive Neuropathic Pain Responses by Modulating Gliosis and CCL2-CCR2/CX3CL1-CX3CR1 Signaling in Spinal Cord-Injured Female Mice

Anna Bagó-Mas [1,2], Andrea Korimová [2], Karolína Bretová [2], Meritxell Deulofeu [1], Enrique Verdú [1], Núria Fiol [3], Petr Dubový [2,*] and Pere Boadas-Vaello [1,*]

[1] Research Group of Clinical Anatomy, Embryology and Neuroscience (NEOMA), Department of Medical Sciences, University of Girona, 17071 Girona, Catalonia, Spain; anna.bago@uvic.cat (A.B.-M.); meritxell.deulofeu@hipra.com (M.D.); enric.verdu@udg.edu (E.V.)

[2] Division of Neuroanatomy, Department of Anatomy, Faculty of Medicine, Masaryk University, 62500 Brno, Czech Republic; andrea.korimova@med.muni.cz (A.K.); karolina.bretova@med.muni.cz (K.B.)

[3] Department of Chemical Engineering, Agriculture and Food Technology, Polytechnic School, University of Girona, 17003 Girona, Catalonia, Spain; nuria.fiol@udg.edu

* Correspondence: pdubovy@med.muni.cz (P.D.); pere.boadas@udg.edu (P.B.-V.)

Supplementary Figure S1. Original scanned full blots for GFAP, IBA1, CCL2, CCR2, CX3CL1, CX3CR1 and CatS in dlPAG (D) and vlPAG (V) in figures 6 and 7.

D1: dlPAG sham; **D2**: dlPAG SCI+saline; **D3**: dlPAG SCI+GSE15; **D4**: dlPAG SCI+CE10; **V1**: vlPAG sham; **V2**: vlPAG SCI+saline; **V3**: vlPAG SCI+GSE15; **V4**: vlPAG SCI+CE10;

D1-D4_V1-V41_GFAP

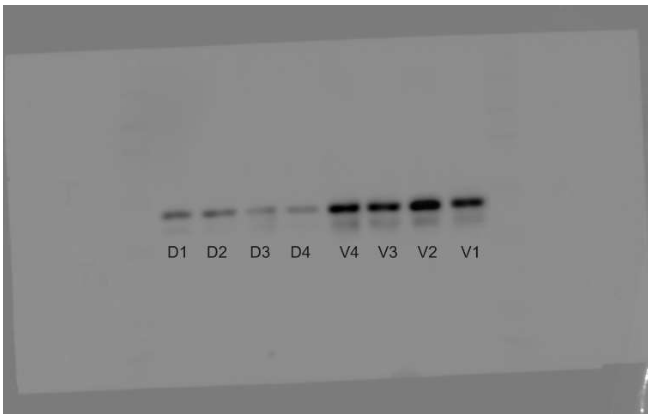

D1-D4_V1-V41_Iba1

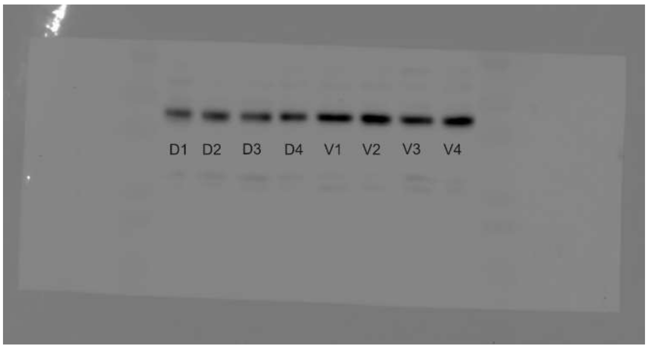

D1-D4_V1-V41_CCL2

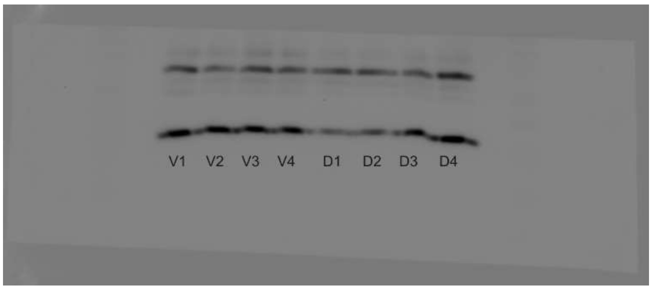

D1-D4_V1-V41_CCR2

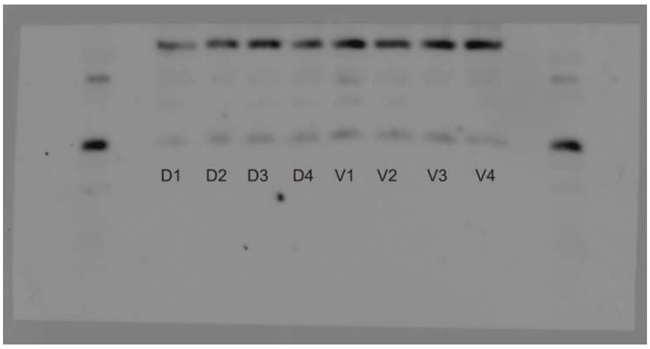

D1-D4_V1-V41_CX3CL1

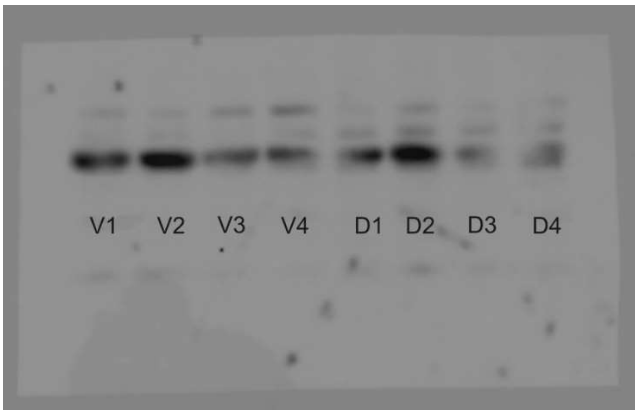

D1-D4_V1-V41_CX3CR1

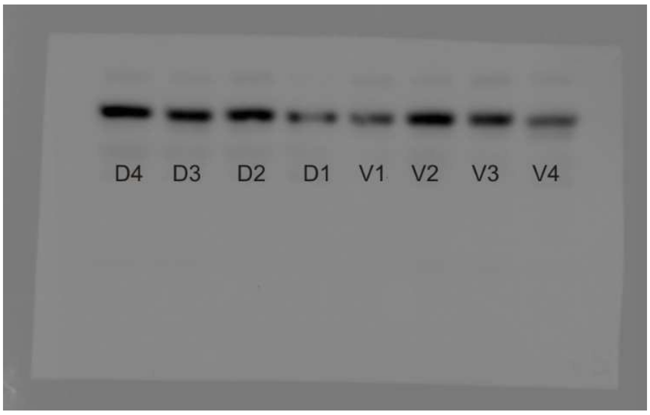

D1-D4_V1-V4_CatS

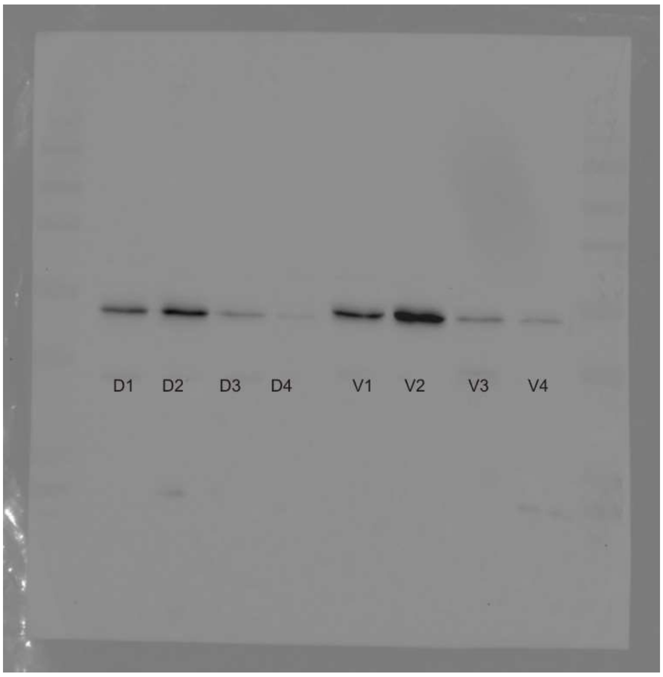

Supplementary Figure S2. Original scanned full blots for GFAP, IBA1, CCL2, CCR2, CX3CL1, CX3CR1 and CatS in RVM in **figure 8**

R1: RVM sham; **R2**: RVM SCI+saline; **R3**: RVM SCI+GSE15; **R4**: RVM SCI+CE10;

R1-R4_GFAP

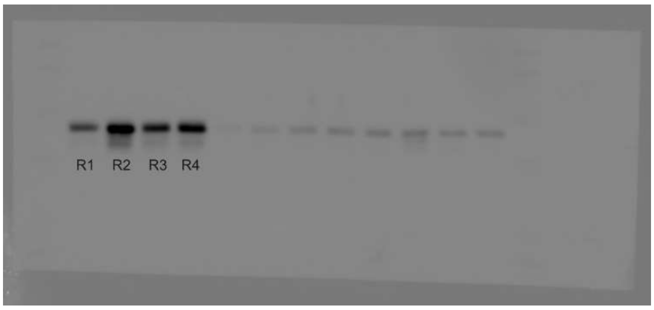

R1-R4_Iba1

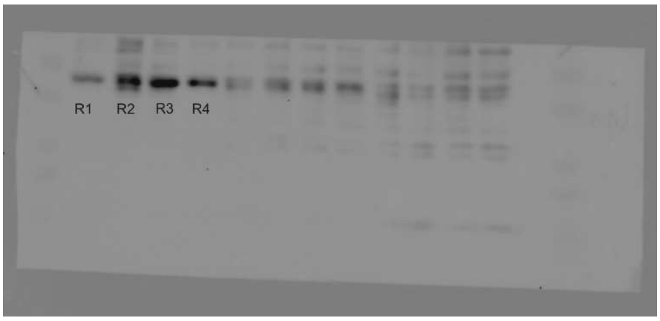

R1-R4_CCL2

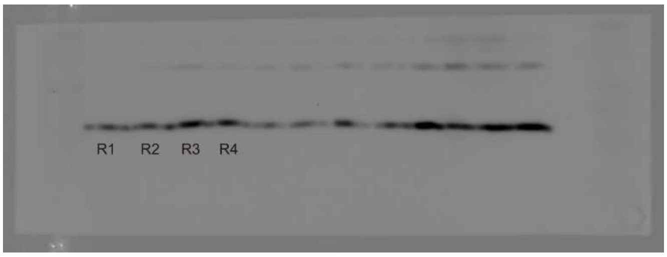

R1-R4_CCR2

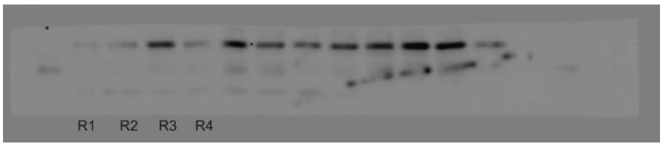

R1-R4_CX3CL1

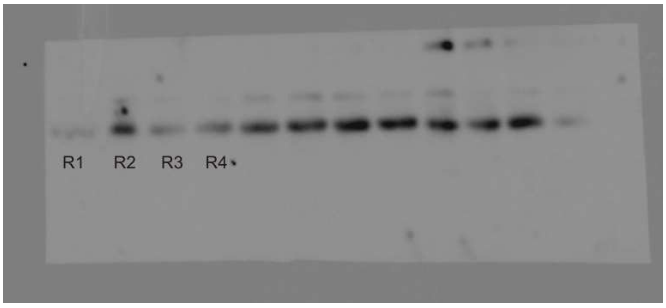

R1-R4_CX3CR1

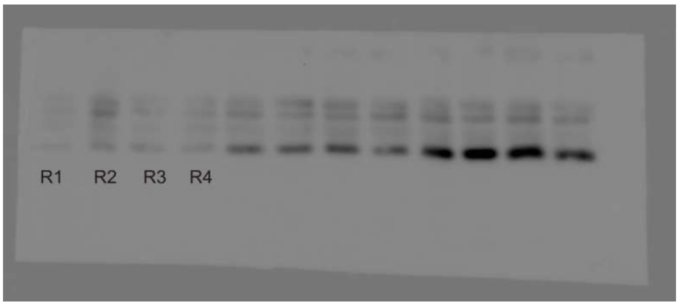

R1-R4_CatS

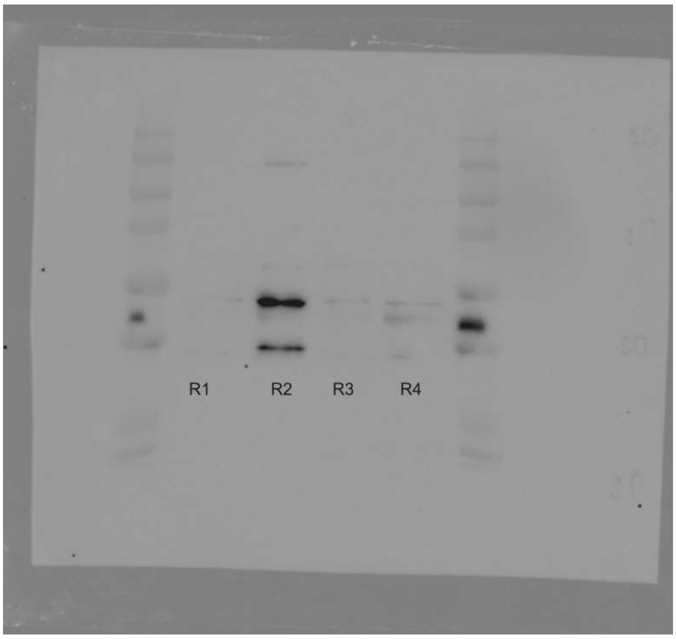

Supplementary Figure S3. Original scanned full blots for GFAP, IBA1, CCL2, CCR2, CX3CL1, CX3CR1 and CatS in ACC in **figure 9**

ACC1: ACC sham; **ACC2**: ACC SCI+saline; **ACC3**: ACC SCI+GSE15; **ACC4**: ACC SCI+CE10;

ACC_GFAP

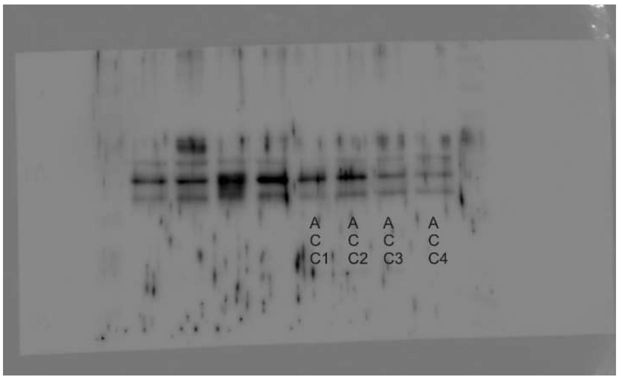

ACC_Iba1

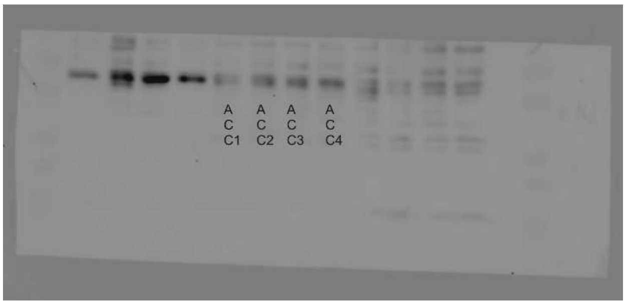

ACC_CCL2

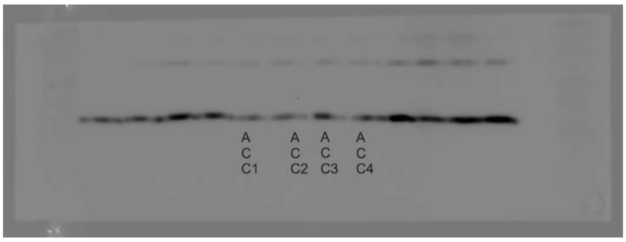

ACC_CCR2

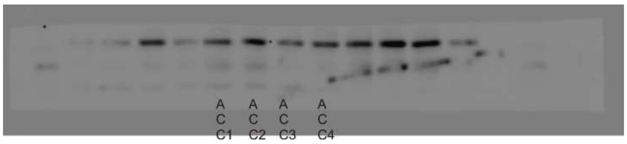

ACC_CX3CL1

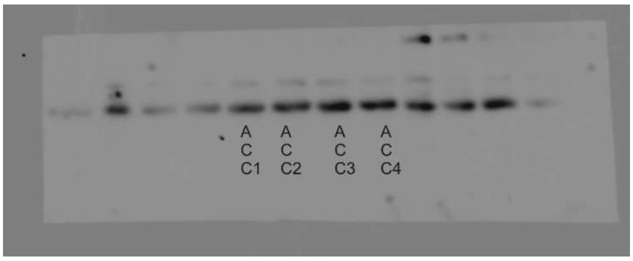

ACC_CX3CR1

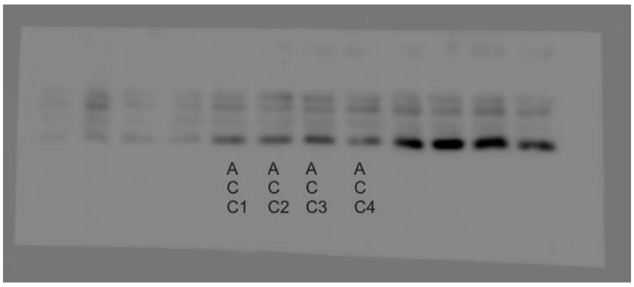

ACC_CatS

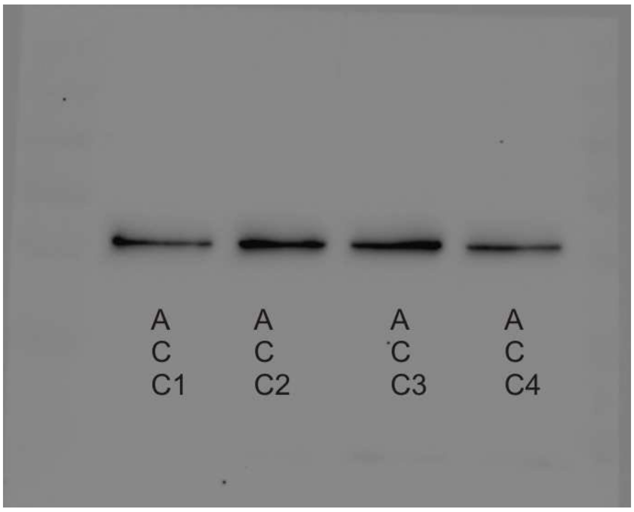

Supplementary Figure S4. Original scanned full blots for GFAP, IBA1, CCL2, CCR2, CX3CL1, CX3CR1 and CatS in AMG in **figure 10**

AMG1: AMG sham; **AMG2**: AMG SCI+saline; **AMG3**: AMG SCI+GSE15; **AMG4**: AMG SCI+CE10;

AMG_GFAP

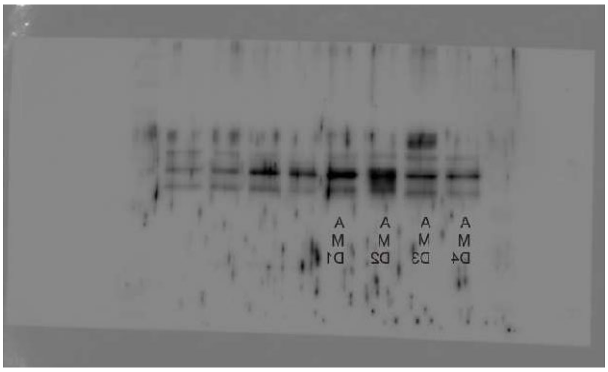

AMG_Iba1

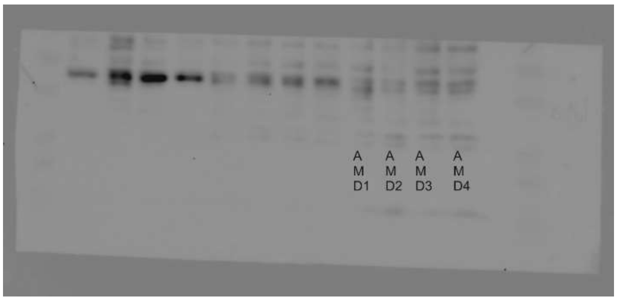

AMG_CCL2

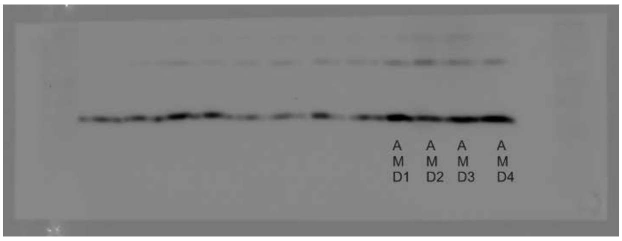

AMG_CCR2

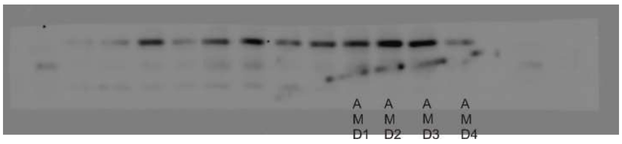

AMG_CX3CL1

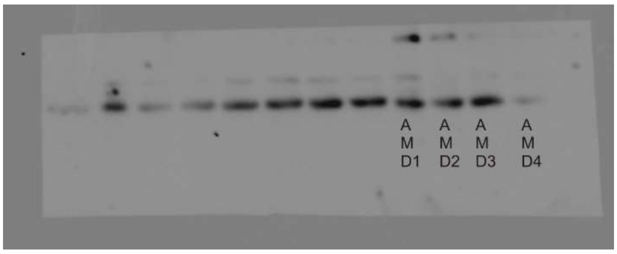

AMG_CX3CR1

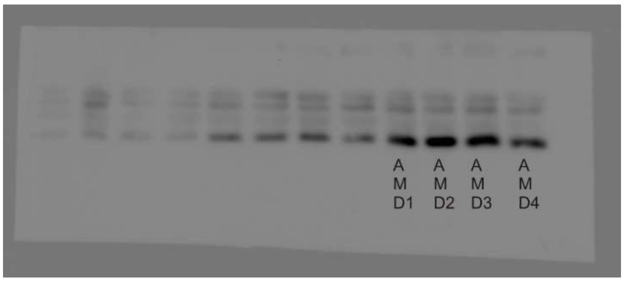

AMG_CatS

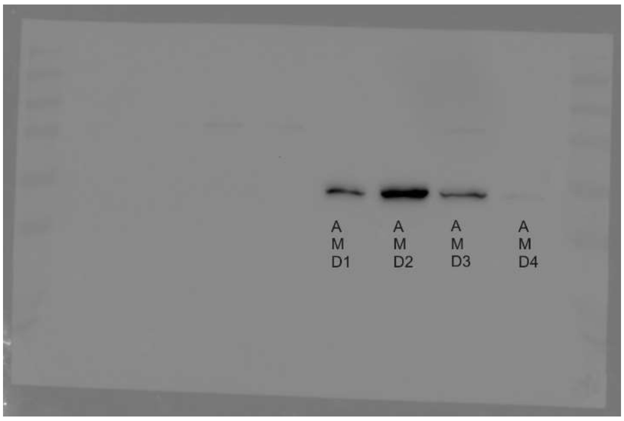

Supplement: Supplementary file 1 [file ijms-26-03325-s001.zip › ijms-3555782-supplementary.pdf]
